# Unraveling the Catalyst‐Solvent Interactions in Lean‐Electrolyte Sulfur Reduction Electrocatalysis for Li−S Batteries

**DOI:** 10.1002/anie.202213863

**Published:** 2022-11-22

**Authors:** Huan Li, Rongwei Meng, Yong Guo, Chao Ye, Debin Kong, Bernt Johannessen, Mietek Jaroniec, Shi‐Zhang Qiao

**Affiliations:** ^1^ School of Chemical Engineering & Advanced Materials The University of Adelaide Adelaide SA 5005 Australia; ^2^ School of Chemical Engineering and Technology Tianjin University Tianjin 300350 China; ^3^ College of New Energy China University of Petroleum (East China) Qingdao 266580 China; ^4^ Australian Synchrotron, ANSTO 800 Blackburn Rd. Clayton VIC 3168 Australia; ^5^ Department of Chemistry and Biochemistry & Advanced Materials and Liquid Crystal Institute Kent State University Kent OH 44242 USA

**Keywords:** Catalyst-Solvent Interactions, Electrocatalysis, Lean Electrolyte Condition, Li−S Batteries, Sulfur Reduction Reaction

## Abstract

Efficient catalyst design is important for lean‐electrolyte sulfur reduction in Li−S batteries. However, most of the reported catalysts were focused on catalyst‐polysulfide interactions, and generally exhibit high activity only with a large excess of electrolyte. Herein, we proposed a general rule to boost lean‐electrolyte sulfur reduction by controlling the catalyst‐solvent interactions. As evidenced by synchrotron‐based analysis, in situ spectroscopy and theoretical computations, strong catalyst‐solvent interaction greatly enhances the lean‐electrolyte catalytic activity and battery stability. Benefitting from the strong interaction between solvent and cobalt catalyst, the Li−S battery achieves stable cycling with only 0.22 % capacity decay per cycle with a low electrolyte/sulfur mass ratio of 4.2. The lean‐electrolyte battery delivers 79 % capacity retention compared with the battery with flooded electrolyte, which is the highest among the reported lean‐electrolyte Li−S batteries.

## Graphical abstract

The catalyst‐solvent interaction is found to determine the lean‐electrolyte sulfur reduction activity and cyclic stability for Li−S batteries. This interaction is confirmed via a series of synchrotron‐based analysis, in situ spectroscopy and theoretical computations. Benefitting from the strong interaction between solvent and cobalt catalyst, the Li−S battery achieves stable cycling and high capacity retention under lean electrolyte conditions.

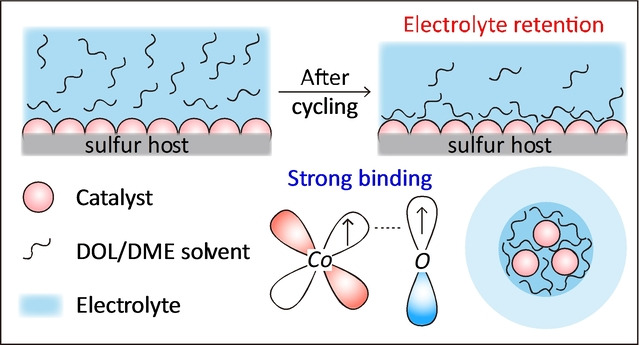

## Introduction

The practical energy density of Li−S battery still remains far from its theoretical value due to excessive use of electrolytes.^[1]^ The electrolyte occupies a large percentage of weight and volume among the whole device, decreasing the actual energy density.[^1a^, ^1b^] Therefore, it is important to reduce electrolyte dosage for Li−S batteries. However under lean‐electrolyte conditions, the sulfur cathode usually undergoes sluggish reduction kinetics, leading to a low output capacity of battery.^[2]^ This demands efficient catalyst design to promote lean‐electrolyte sulfur reduction kinetics. However, most of the reported electrocatalysts exhibit high activity when a large excess of electrolyte is used.^[3]^ The design of catalysts working well under lean electrolyte conditions is still lacking. Although some catalysts were proposed to facilitate sulfur reduction in lean electrolyte,^[4]^ it has been unnoticed that the operation of Li−S battery is concomitant with electrolyte consumption. The battery gradually runs off electrolyte followed by capacity decay, finally leading to battery failure.^[5]^ These problems are exacerbated especially when the usage of electrolyte decreases. Therefore, a general design rule of catalysts is needed to boost the lean‐electrolyte sulfur reduction activity and simultaneously restrain electrolyte loss to increase cycling stability.

Sulfur reduction reaction (SRR) undergoes consecutive reduction from sulfur to polysulfides, and then from polysulfides to sulfide. The conversion efficiency of polysulfide intermediates determines the energy output during SRR.[^1i^, ^6^] These polysulfides are solvated by solvent molecules and are electrochemically reduced on the surface of catalyst.^[7]^ Therefore, the local solvent environment of catalyst is critical for polysulfide conversions, which is determined by solvent affinity toward catalysts. Ether solvents are mostly used in Li−S batteries, for example, 1,3‐dioxolane (DOL), 1,2‐dimethoxyethane (DME).[^1a^, ^1b^, ^2b^] The oxygen atoms in these solvent molecules expose 2p‐orbital electrons, which tend to hybridize with d‐orbital of metal catalysts.^[8]^ During the operation of Li−S batteries, the shuttle of solvated polysulfides from cathode to anode leads to the consumption of both active sulfur species and solvent molecules. The reported solutions to this problem are often based on the control of catalyst‐polysulfide interactions.[^1a^, ^1h^, ^2b^, ^2c^] However, the catalyst‐solvent interactions for lean‐electrolyte Li−S batteries have not been studied. The catalyst surface with more adsorbed solvent molecules is expected to promote polysulfide conversion and restrain electrolyte loss. Therefore it is essential to unravel the role of local solvent environments on the catalyst surface for lean‐electrolyte sulfur reduction.

In this work, we present a general rule to boost lean‐electrolyte sulfur reduction by controlling the catalyst‐solvent interactions. Co, Rh, Pt are selected as comparative model catalysts because these metals are typical 3d, 4d and 5d catalysts which are chemically stable during SRR.^[9]^ It is found that the strength of catalyst‐solvent interaction plays a decisive role on lean‐electrolyte SRR activity, electrolyte consumption and battery stability. Synchrotron‐based X‐ray adsorption fine structures are used to confirm metal‐oxygen binding between catalyst and solvent molecules. Theoretical computation further reveals that lower occupancy of anti‐bonding O 2p orbital electron states of adsorbed solvent results in the stronger interaction. Compared to Rh and Pt catalysts, the greater SRR activity of Co catalyst is only obvious under lean electrolyte conditions, including higher kinetic current, lower Tafel slope and more Li_2_S deposition. As a result, the Li−S battery exhibits stable cycling while maintaining a high lean‐electrolyte capacity with low electrolyte consumption.

## Results and Discussion

### Consumption of Solvents and Demonstration of Catalyst‐Solvent Binding

**Figure 1:**
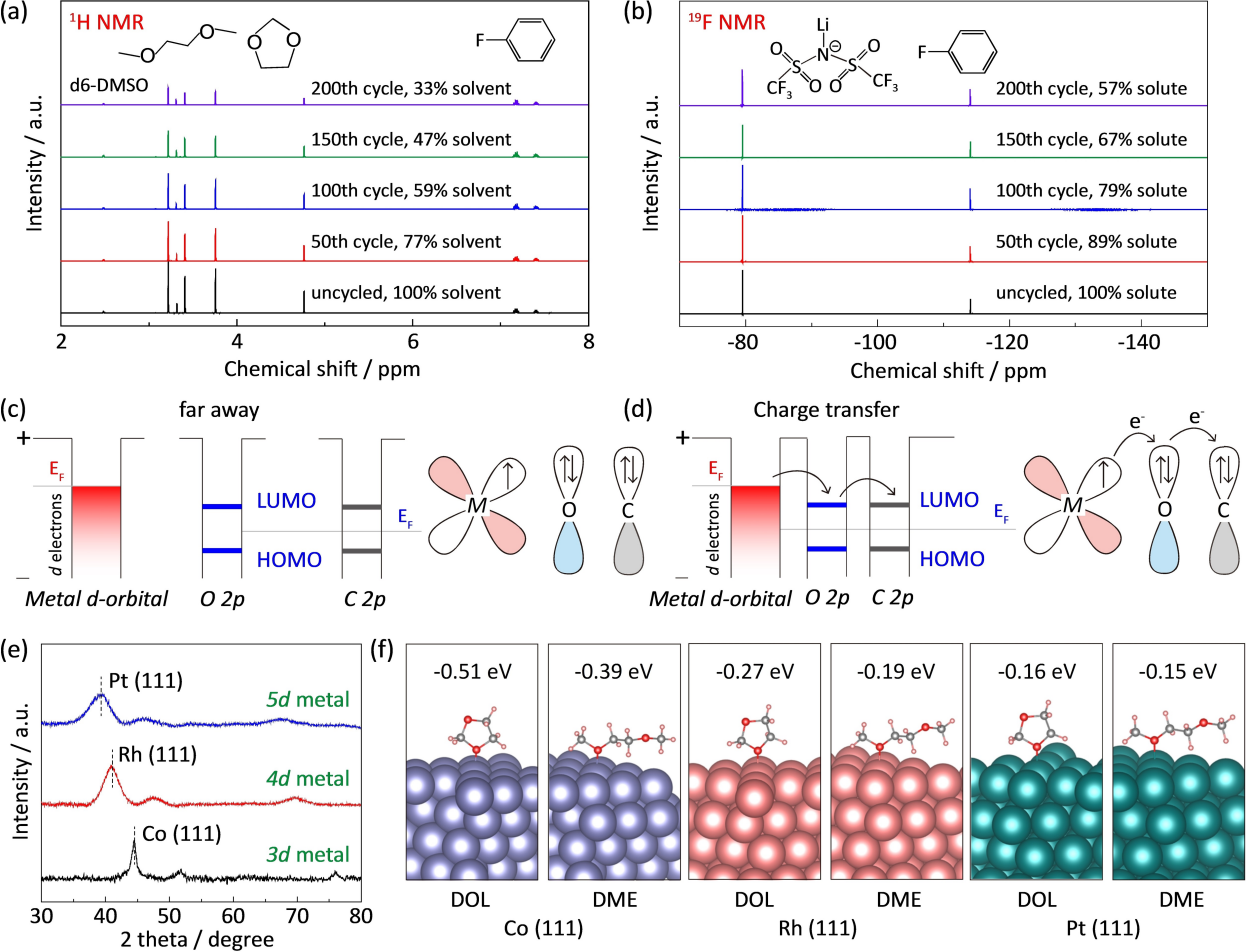
Consumption of solvents and demonstration of catalyst‐solvent interactions. a) ^1^H and b) ^19^F NMR spectra of electrolytes extracted from Li−S batteries at different cycles. ^1^H spectra are used to quantify the content of DOL/DME solvent, and ^19^F spectra are used to determine the amounts of LiTfSI solute. A known amount of fluorobenzene (0.1 M) is used as internal reference, and its peak area is normalized to 100 %; Scheme of the energy levels of a metal (left) and a solvent molecule (right): c) When they are far away from each other; d) Charge transfer; e) XRD patterns of prepared Co, Rh, Pt metal catalysts on graphene substrates; f) Optimized models of DOL/DME solvent molecules on the surface of Co (111), Rh (111) and Pt (111).

The electrolyte consumption during cycling of Li−S battery was quantified by nuclear magnetic resonance (NMR) spectra (Figure S1).[^5a^, ^10^] ^1^H and ^19^F NMR spectra were respectively investigated for solvents and solute, because H and F signals come exclusively from the DOL/DME solvent and Lithium bis(trifluoromethane sulfonimide) solute (LiTfSI) (Figure S2). The electrolyte consumption is mainly caused by polysulfide shuttle and anode corrosion. As shown in Figures 1a and b, the contents of both solvent and solute gradually decrease during battery cycling. Only 33 % of solvent remains after 200 cycles, in contrast 57 % of original content of lithium salt solute is retained. This indicates the significant consumption of solvent during battery cycling, leading to the dramatic capacity decay and battery failure. Therefore, it is essential to restrain the solvent loss in Li−S batteries.

Considering that the oxygen 2p‐orbital electrons among the solvent molecules are exposed to the outer shell, these electrons can be effectively used to bind these solvent molecules with metal catalysts through d‐2p hybridization. Figure 1c shows a schematic representation of the energy levels of a metal and a typical solvent molecule when they are separated from each other. When the solvent molecule contacts with the metal, the outer‐shell p‐orbital electrons of oxygen become more reactive and O−C bonds are impaired. The d‐orbital electrons on the metal surface transfer through the unoccupied energy level of O 2p‐orbital of solvent molecule, and finally are accumulated on the adjacent carbon atom (Figure 1d and S3). As a result, the electronic density of solvent rearranges due to the charge transfer at the interface, forming metal‐ oxygen bond between metal catalysts and solvent molecules.

To investigate the binding strength of metal‐oxygen bonds, typical 3d, 4d, 5d nanometal catalysts of Co, Rh, Pt are comparatively synthesized with dominating (111) crystal planes as evidenced by the X‐ray diffraction patterns (Figure 1e and S4). Theoretical computations based on density functional theory (DFT) are used to model the interactions of DOL/DME molecules on the metal (111) surfaces. As shown in Figure 1f, the DOL and DME molecules prefer to be adsorbed on the metal surfaces via vertical orientations (Figure S5). All these metals bind with solvent molecules via metal‐oxygen bonds, confirming the efficient catalyst‐solvent binding at the interface. Co metal catalyst exhibits much stronger binding with DOL and DME molecules compared to those on Rh and Pt catalysts (Figure S6). Therefore, Co metal catalyst is expected to demonstrate greater activity than Rh and Pt under lean electrolyte conditions.

### Spectroscopic Confirmation of Catalyst‐Solvent Interaction

**Figure 2:**
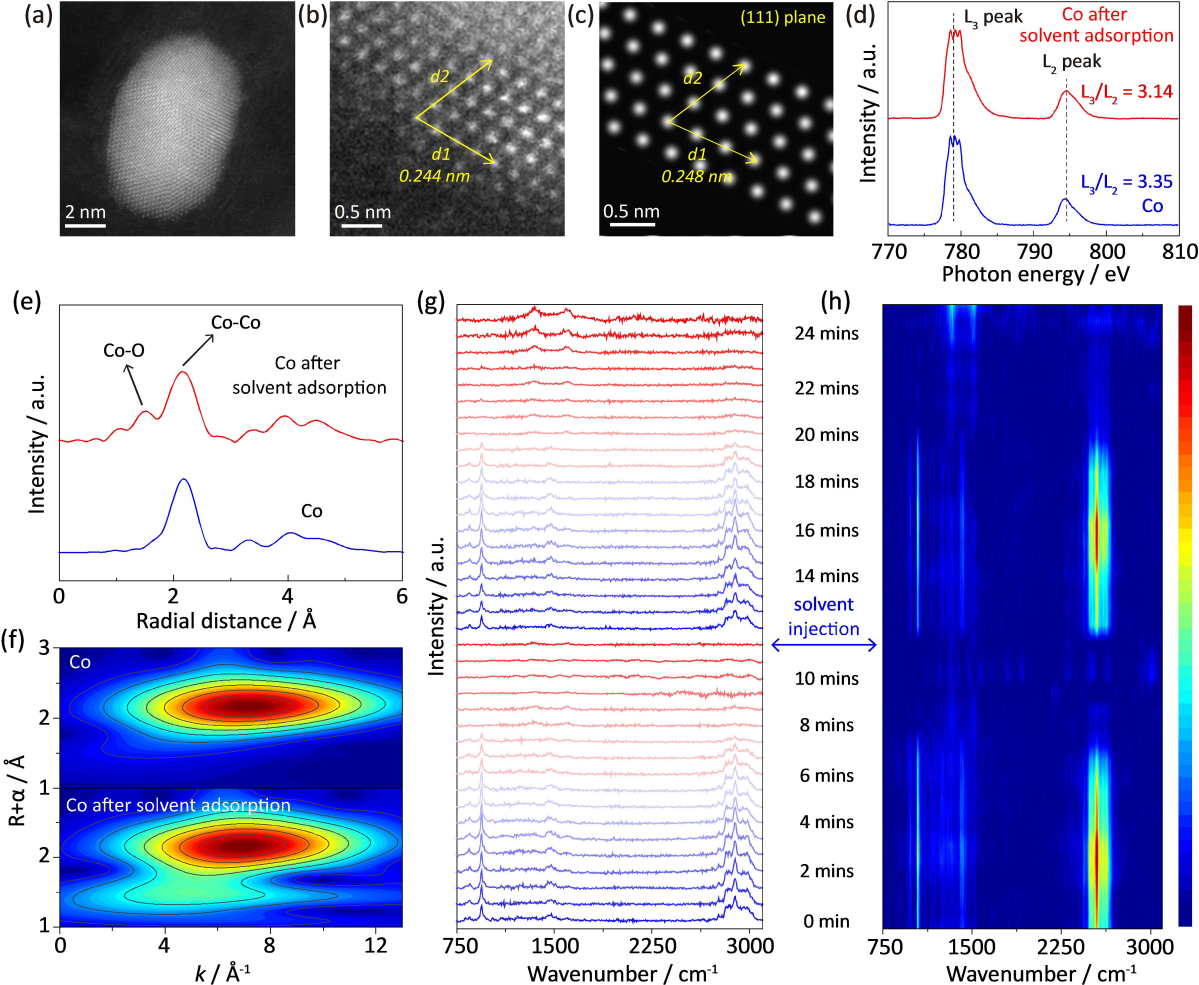
Spectroscopic confirmation of catalyst‐solvent interaction. a, b) High‐resolution TEM images of Co catalyst; c) Simulated image of Co (111) plane that coincides well with the experimental observations; d) Co L‐edge NEXAFS spectra and e) Co K‐edge EXAFS spectra with f) corresponding wave‐transform patterns of pristine Co catalyst and after solvent adsorption; In situ Raman spectra revealing the local solvent environment on Co surface: g) Time‐dependent stacked plots and h) contour pattern.

To experimentally unravel the catalyst‐solvent binding, the synchrotron based near‐edge X‐ray adsorption fine‐structure (NEXAFS), extended X‐ray adsorption fine structure (EXAFS), and in situ Raman spectra were used. As shown in Figure 2a and S7, Co nanoparticles are uniformly distributed on the 3D graphene network. The high‐resolution transmission microscopy (TEM) image confirms the dominated (111) crystal plane (Figures 2b and c). The content of Co is 14.2 wt % on graphene substrate (Figure S8) and loading of other metal nanoparticles for Rh and Pt does not alter the properties of graphene (Figure S9). The Co catalyst presents excellent properties toward surface wetting with solvents as suggested by the contact angles (Figure S10). Figure 2d shows the NEXAFS spectra of pristine Co catalyst and Co after solvent adsorption. The L_3_/L_2_ intensity decreases from 3.35 to 3.14 after solvent adsorption, indicating a higher valence of Co while binding with DOL/DME molecules (Figure S11).^[11]^ The Co K‐edge EXAFS spectra are used to reveal the origin of higher valence of Co catalyst after solvent adsorption. As shown in Figure 2e, an obvious scattering radial distance of ≈1.8 Å is observed after adsorption of solvent, which belongs to the Co−O bond between catalyst and solvent molecules (Figures S12 and S13). The wavelet‐transform contour in Figure 2f confirms the Co−O bond by comparing with the spectrum of the CoO standard reference (Figure S14). The X‐ray photoelectron spectra (XPS) coincide well with the NEXAFS and EXAFS analysis (Figure S15). In situ Raman spectra were used to characterize the local solvent environment and solvent retention on the catalyst surface.^[12]^ The Co catalyst was loaded in an open cell with DOL/DME. Due to the volatile nature of DOL and DME, these solvent molecules tend to evaporate from the material surface. Figure 2g presents the time‐dependent Raman spectra on the surface of Co catalyst with DOL/DME adsorption, and its corresponding contour pattern is shown in Figure 2h. The peaks at ≈940, ≈1460 and ≈2900 cm^−1^ are assigned to DOL and DME molecules (Figure S16). Due to the strong binding between Co catalyst and solvent, the solvent shows a long retention time of ≈7 mins under atmosphere. In contrast, the solvent molecules can only retain for ≈4 mins without Co catalyst (Figure S17). The above results confirm the catalyst‐solvent binding via Co−O bond, and Co catalysts exhibit better affinity and strong adsorption of solvent molecules.

### Quantifying the Solvent Adsorption on Catalyst Surfaces

**Figure 3:**
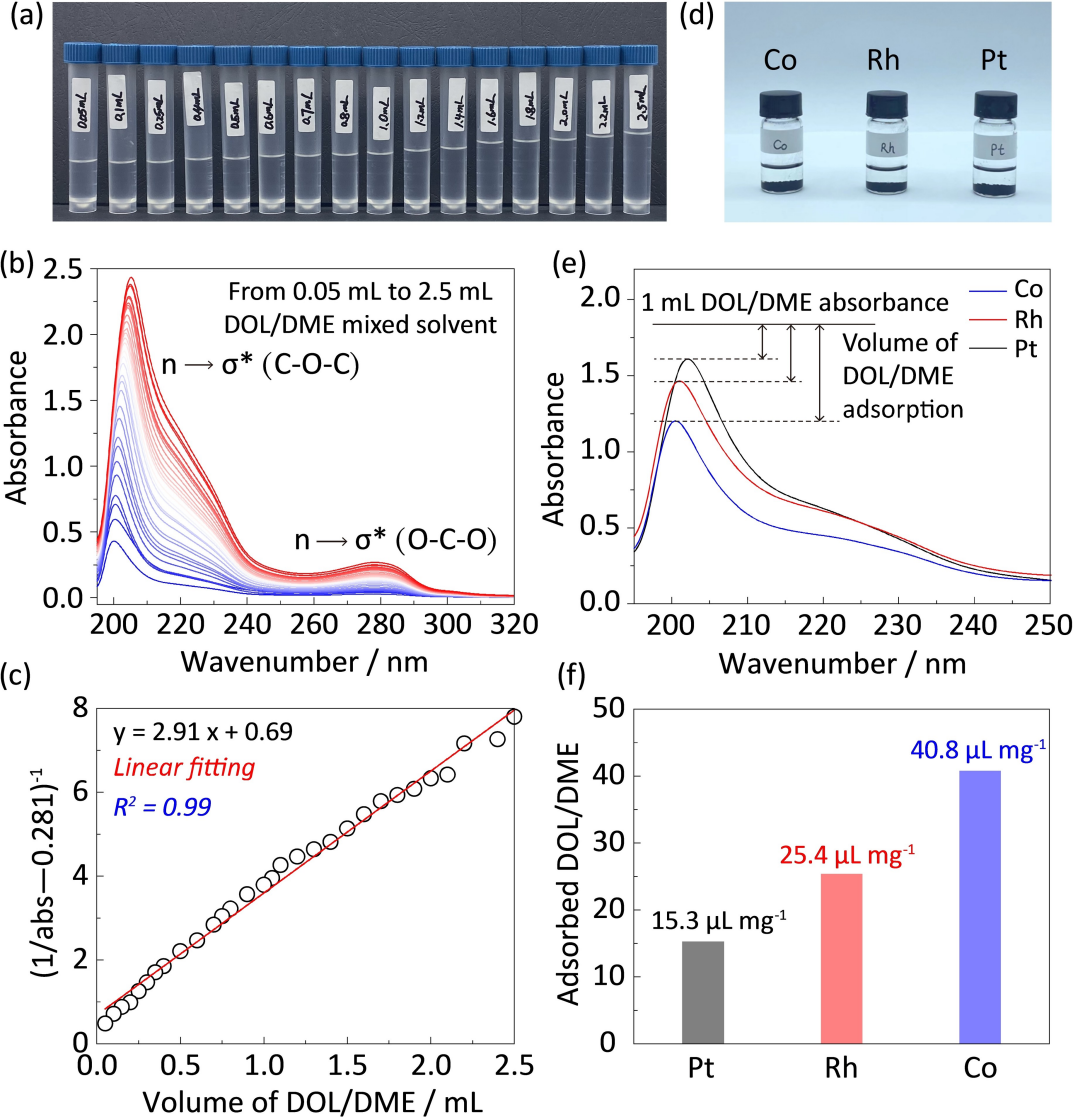
Quantifying the solvent adsorption on catalyst surfaces. a) Digital photograph of standard solutions with 3 mL of ethanol and DOL/DME solvent ranging from 0.05 to 2.5 mL, which are used to plot the standard relation between the solvent volume and UV/Vis absorbance; b) The stacked UV/Vis plots of solutions in (a); c) Linear fitting between the UV/Vis absorbance and DOL/DME volume; d) Digital photograph of Co, Rh and Pt catalysts immersed in DOL/DME solvent. The supernatant is used to quantify the solvent adsorption; e) UV/Vis plots for 1 mL of DOL/DME solvent on Co, Rh and Pt catalysts; f) The quantified adsorbed volume of solvent for Co, Rh and Pt catalysts.

Both DOL and DME are ether molecules, and the C−O bonds demonstrate obvious absorbance in the ultraviolet region.^[13]^ Therefore, ultraviolet‐visible spectroscopy (UV/Vis) is a good method to quantify the solvent adsorption on different catalysts.^[7b]^ Prior to the quantification, a standard plot that correlates the UV/Vis absorbance and DOL/DME volume should be made. Ethanol was selected to dissolve DOL/DME because ethanol shows different UV/Vis peak positions and weak peak intensity as compared to DOL/DME (Figure S18). Figure 3a shows the digital photograph of standard solutions with 3 mL of ethanol and DOL/DME solvent ranging from 0.05 to 2.5 mL (Figure S19). The corresponding UV/Vis curves are shown in Figure 3b. Peaks at ≈205 and ≈280 nm are assigned to the C−O bonds in DOL/DME, which are caused by the electron transition to anti‐bonding σ* orbital (Figure S20). The dominated peak at ≈205 nm is used to plot the standard relation between the solvent volume and UV absorbance. As a result, a well‐fitted linear scaling relationship is seen in Figure 3c, allowing to obtain the specified solvent volumes at different UV/Vis absorbance (Figures S21 and S22). To compare the adsorption ability of catalysts, the Co, Rh, Pt catalysts were immersed in DOL/DME solvent (Figure 3d). After adsorption, 1 mL of supernatant was taken out and mixed with 3 mL of ethanol for UV/Vis test. As shown in Figure 3e, the supernatant after adsorption on Co exhibits the lowest peak intensity around ≈205 nm, confirming the strongest interaction of Co with solvent molecules. As a result, Co catalyst is quantified with solvent adsorption of 40.8 μL mg^−1^, which is much higher than those of 25.4 and 15.3 μL mg^−1^ for Rh and Pt catalysts, respectively (Figure 3f).

### Lean‐Electrolyte Sulfur Reduction Electrocatalysis

**Figure 4:**
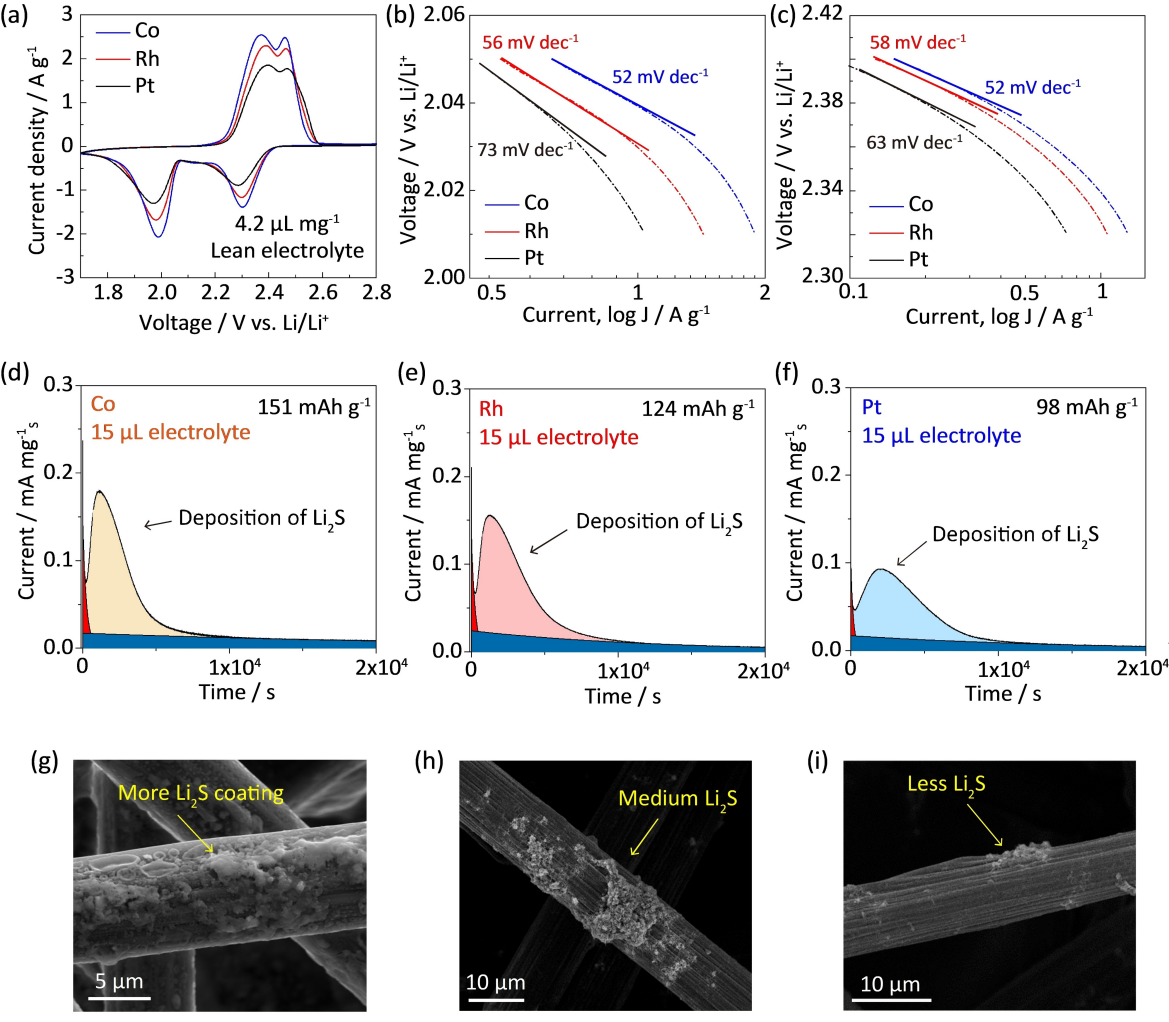
Lean‐electrolyte sulfur reduction electrocatalysis. a) CV curves of Li−S batteries with Co, Rh and Pt catalysts at 0.2 mV s^−1^ under lean electrolyte conditions (E/S=4.2); Tafel plots for b) the electrocatalytic reduction from polysulfides to Li_2_S and c) from sulfur to polysulfides; Potential‐static discharge curves for Li_2_S deposition from polysulfides in 15 μL of lean electrolyte with d) Co, e) Rh and f) Pt catalysts; SEM images of Li_2_S precipitation on carbon fibre using g) Co, h) Rh and i) Pt catalysts.

To investigate the SRR activity under lean‐electrolyte conditions, the cyclic voltammetry (CV) and potential‐static Li_2_S deposition were comparatively tested for Co, Rh and Pt catalysts. Figure 4a compares the CV curves of Li−S batteries with Co, Rh and Pt catalysts at 0.2 mV s^−1^, which were tested with an electrolyte to sulfur (E/S) ratio of 4.2 μL mg^−1^. Two distinct peaks around 2.3 V and 2.05 V are attributed to the reduction from sulfur to polysulfides, and from polysufides to Li_2_S. Co presents a much higher kinetic current for Li_2_S deposition of ≈2.0 A g^−1^ even with lean electrolyte. This suggests its superior catalytic activity for lean‐electrolyte sulfur reduction (Figure S23). Figures 4b and c present the Tafel plots of two reduction ranges for these catalysts. For the reduction from Li_2_S_4_ to Li_2_S, Co exhibits the lowest Tafel slope of 52 mV dec^−1^ and the lowest overpotential of 110 mV (by considering 2.15 V as the equilibrium potential). These results confirm the higher electrocatalytic activity of Co under lean electrolyte conditions.

To confirm higher catalytic activity of Co catalysts to regulate Li_2_S precipitation with lean electrolyte, the potential‐static discharge was carried out by loading Co, Rh, Pt catalysts on carbon fiber paper with only 15 μL electrolyte addition.^[14]^ The Co electrode exhibited a significantly greater Li_2_S precipitation capacity of 151 mAh g^−1^ than Rh and Pt with 124 mAh g^−1^ and 98 mAh g^−1^, respectively, Figures 4d–f. Obviously, Li_2_S nucleates and precipitates are seen on the Co‐contained cathode surface in the scanning electron microscopy (SEM) image (Figure 4g), which are more pronounced than those on the cathode surfaces with Rh and Pt catalysts (Figures 4h and i). These findings confirm higher activity of Co to catalyze polysulfides into Li_2_S products under lean‐electrolyte conditions.

### Mechanistic Insights into Catalyst‐Solvent Interaction

**Figure 5:**
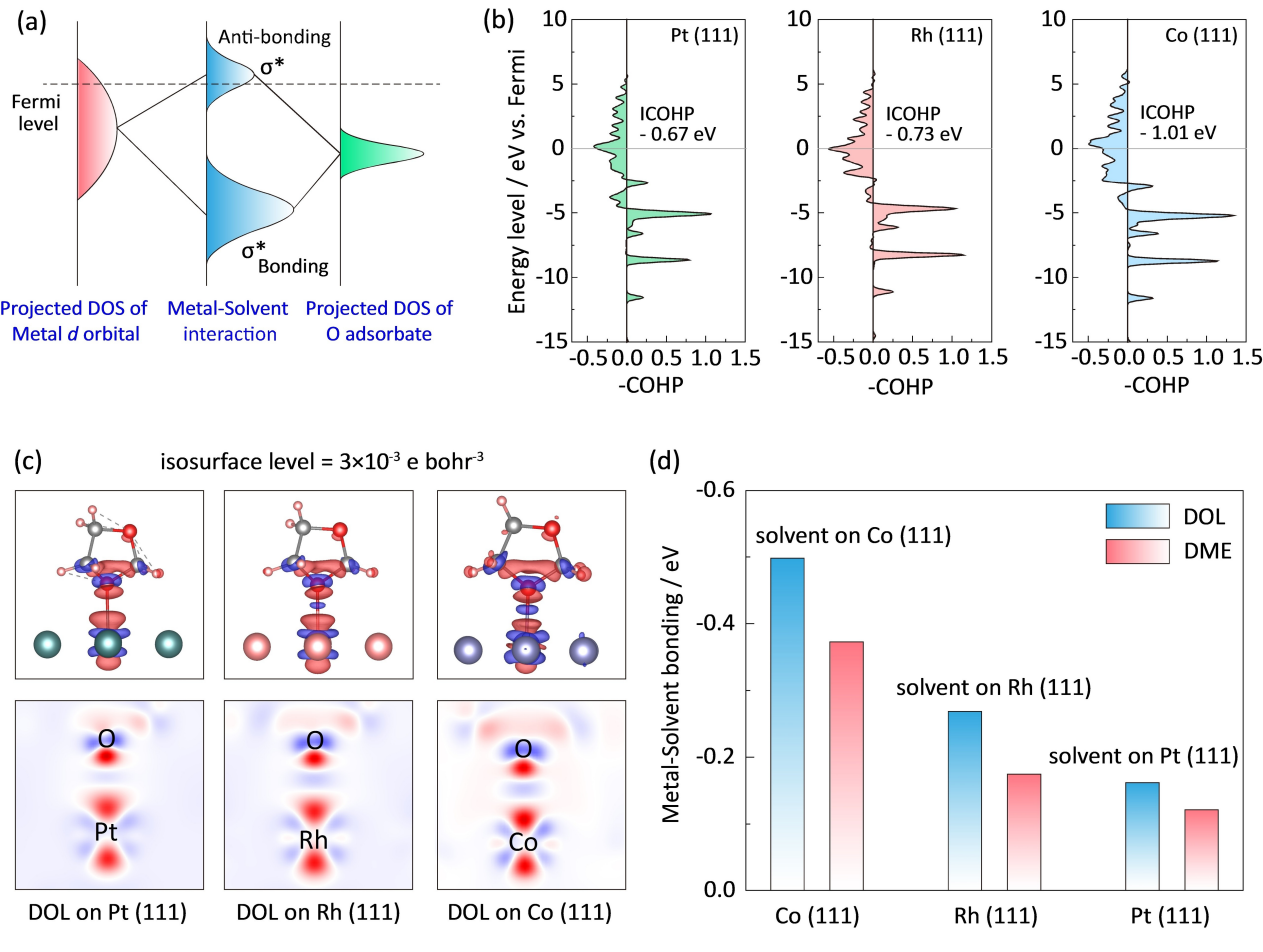
Mechanistic insights into catalyst‐solvent interactions. a) Energy level diagram showing orbital hybridization of metal active sites and solvent adsorbate. σ and σ* indicate bonding and anti‐bonding states, respectively; b) Projected crystal orbital Hamilton population (pCOHP) between the surface metal atom and the oxygen atom of DOL molecule. Green filling, Pt; Pink filling, Rh; Blue filling, Co. The insets present the integrated COHP (ICOHP) values; c) Charge‐transfer maps of DOL molecules on Pt (111), Rh (111) and Co (111). Red indicates electron accumulation and blue denotes electron depletion; d) Summary of binding strength of DOL and DME molecules on different metal surfaces.

The relationship between the adsorption ability of a material and its electronic structure can be schematically explained by the underlying as illustrated in Figure 5a.^[15]^ When a solvent molecule from the electrolyte is adsorbed on the metal surface to form metal‐oxygen bond, the electronic states of the metal interact with those of oxygen. Consequently, the hybridized energy levels split into two groups: one is the anti‐bonding orbital (σ*) close to Fermi level (E_F_), the other is the bonding orbital (σ) positioned far from Fermi level. The difference in the adsorption strength comes both from bonding states and anti‐bonding states. A higher occupancy of bonding states and a lower occupancy of anti‐bonding states result in a stronger solvent‐metal catalyst interaction. In this work, we introduced projected crystal orbital Hamilton population (pCOHP) to analyze the interaction between the metal catalysts and solvent molecules. We follow the usual way of displaying COHP, namely, drawing bonding contributions to the right and anti‐bonding contribution to the left. As shown in Figure 5b, the filling of bonding orbital populations increases from Pt, Rh to Co but the filling of anti‐bonding orbital populations decreases. This explains the stronger interaction between Co catalysts and solvent molecules. In addition, we calculated the integrated COHP (ICOHP) by calculating the energy integral up to the highest occupied bands (below Fermi level, E_F_), which directly gives more quantitative information on the bonding strength. As shown in Figure 5b and Figure S24, the ICOHP between oxygen atom among DOL molecule and metal atom are −1.01, −0.73 and −0.67 eV for Co, Rh and Pt respectively, and a more negative value of Co confirms its stronger binding with solvent molecules.

The binding strength can be also explained by the charge transfer of solvent molecules on metal surfaces. Figure 5c depicts the charge‐transfer patterns of DOL on Pt (111), Rh (111) and Co (111), where red indicates electron accumulation and blue denotes electron depletion. DOL and DME on Co (111) demonstrates more electrons transferred at the interface compared to those on Rh (111) and Pt (111) (Figures 5c, S25 and S26). The charge‐transfer numbers can be further quantified with Bader charge analysis (Figure S27). The surface Co atom shows higher electron depletion of −0.155 while adsorbed with DOL molecule. This depletion is much larger than those of −0.101 and −0.089 for DOL molecule on Rh and Pt. As a result, Co catalyst exhibits the strongest binding with DOL and DME molecules, which are −0.51 and −0.39 eV, respectively. These values are much higher than those on the Rh and Pt surfaces (Figure 5d). These data indicate that the lower occupation of anti‐bonding state of Co and efficient charge transfer result in a strong binding between Co and solvent molecules.

### Lean‐Electrolyte Electrocatalysis in Li−S Batteries

**Figure 6:**
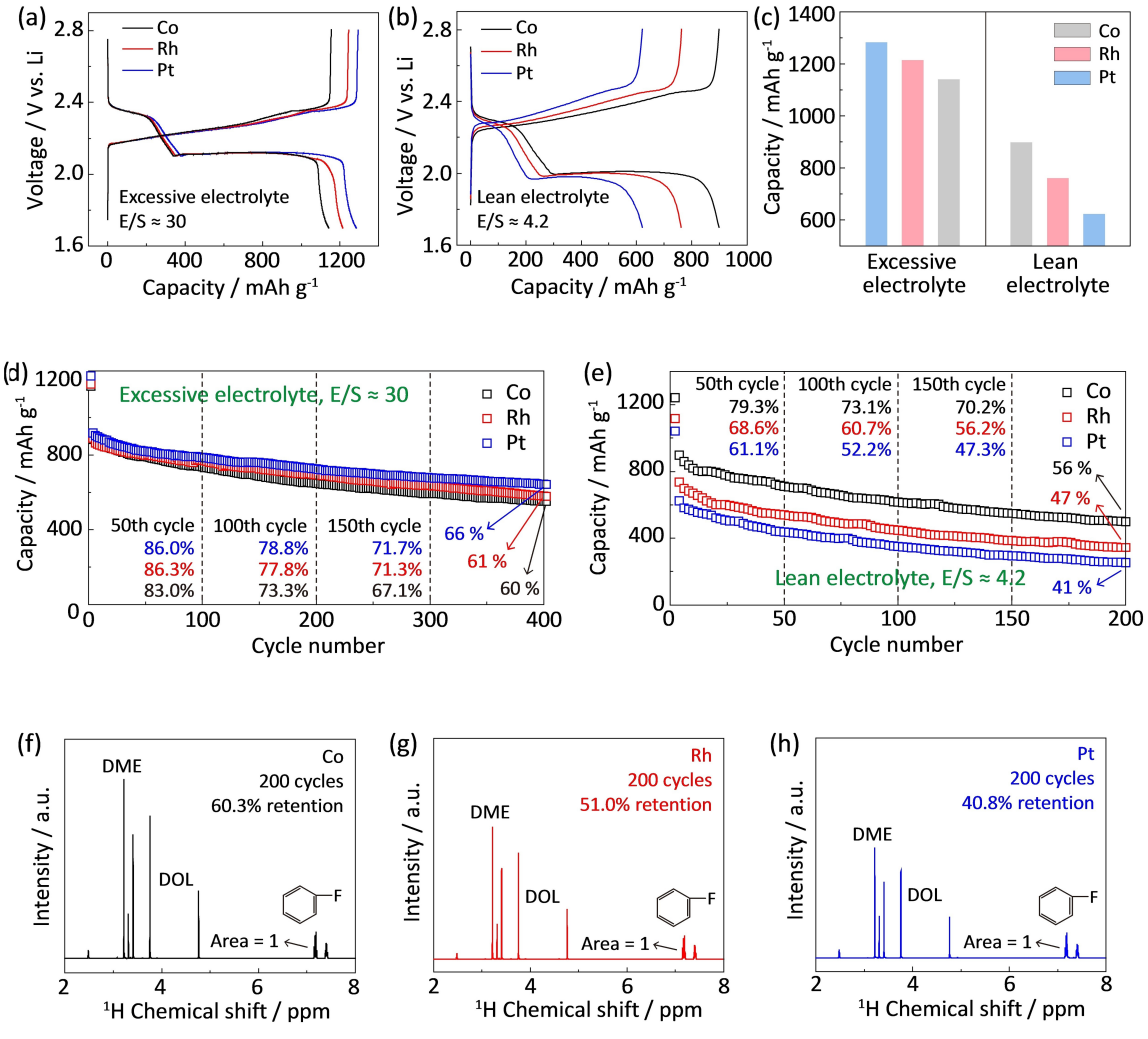
Lean‐electrolyte electrocatalysis in Li−S batteries. Galvanostatic charge‐discharge curves for Li−S batteries using Co, Rh and Pt catalysts at 0.2 C (a) with excessive electrolyte (E/S=30) and sulfur loading of ≈0.5 mg cm^−2^ and b) lean electrolyte (E/S=4.2) and sulfur loading of ≈5 mg cm^−2^; c) Capacity comparison with Co, Rh and Pt catalysts under flooded and lean electrolyte conditions; Cycling performance for Li−S batteries with Co, Rh and Pt catalysts with d) excessive electrolyte (E/S=30) with sulfur loading of ≈0.5 mg cm^−2^ at 1.0 C and e) lean electrolyte (E/S=4.2) with sulfur loading of ≈5 mg cm^−2^ at 0.2 C; f–h) The solvent retention after 200 cycles for Li−S batteries with Co, Rh and Pt catalysts.

To confirm the effect of lean‐electrolyte SRR electrocatalysis on the battery performance, the Li−S batteries with different catalysts were assembled and comparatively tested. Figure 6a presents the galvanostatic charge‐discharge curves for Li−S batteries using Co Rh and Pt catalysts with excessive electrolyte (E/S=30). Co‐catalyzed Li−S battery shows a slightly lower specific capacity of 1144 mAh g^−1^ due to the higher binding energy between polysulfides and Co (Figure S28). Too strong binding energy would lead to a lower catalytic activity following Sabatier's principle.^[1i]^ In contrast, Pt‐catalyzed battery exhibits a higher capacity due to the greater catalytic activity of Pt (Figure S29). However, under lean‐electrolyte conditions (E/S=4.2), the specific capacity of Pt‐catalyzed Li−S battery dramatically decreases to 623 mAh g^−1^, and the Co‐catalyzed battery delivers the highest capacity of 900 mAh g^−1^ (Figure 6b). Therefore, the greater SRR activity of Co, catalyst is only obvious under lean electrolyte conditions. Compared to the battery with flooded electrolyte, the lean‐electrolyte battery with Co catalyst maintains 79 % capacity, which is the highest capacity retention among systems with low electrolyte dosages reported so far (Table S1). However, the lean‐electrolyte battery with Pt catalyst only retains 45 % of its capacity. Therefore, the activity of these catalysts is highly dependent on the electrolyte dosage, and a strong binding between catalyst and solvent contribute a high battery capacity under lean electrolyte conditions (Figure 6c, Figure S30). Additionally, the high lean‐electrolyte catalytic activity of Co can be also confirmed by the lower charge‐discharge overpotentials compared with those for the batteries with Rh and Pt, Figure S31. The cycling performances are also compared with lean electrolyte and flooded electrolyte. Prior to the cycling test, the battery was first charged‐discharged at 0.1 C for pre‐activation, and the following initial capacity is based on the discharge capacity at the second cycle under 0.2 C. Li−S batteries with Co, Rh and Pt catalysts are able to work steadily upon cycles. This suggests that these metal catalysts are electrochemically stable while used as SRR catalysts in Li−S batteries, coinciding with the previous reports.^[9]^ In the excessive electrolyte, these catalysts show similar battery stability with about ≈900 mAh g^−1^ initial capacity and ≈60 % capacity retention after 400 cycles at 1.0 C (Figure 6d). In contrast, the Co catalyst greatly outperforms other catalysts under lean electrolyte conditions (E/S=4.2, Figure 6e). Specially, the battery with Co catalyst exhibits an initial capacity of ≈900 mAh g^−1^ at 0.2 C, much higher than those of 736 mAh g^−1^ and 627 mAh g^−1^ for Rh‐ and Pt‐catalyzed batteries. Additionally, the Co‐catalyzed battery exhibits higher capacity retention during the following cycles. For example, the Co‐catalyzed battery exhibits capacity retentions of 69 %, 61 % and 56 % at the 100th, 150th and 200th cycles, respectively which are much higher than the corresponding values for the batteries with Rh and Pt catalysts. The Coulombic efficiency is near ≈100 % (Figure S32). By comparison, due to the weak binding between Pt and solvent molecules, the battery with Pt catalyst only delivers an initial capacity of ≈627 mAh g^−1^ with 41 % capacity retention.

After cycling of Li−S batteries, the electrolyte was extracted from the cell and used for NMR analysis to quantify the electrolyte consumption. Because of the known amount of an internal fluorobenzene reference (0.1 M), the integrated area of DOL and DME peaks can be used to quantify the electrolyte retention. The battery with Co catalyst retains 65 % of electrolyte after 200 cycles (Figure 6f), which is much higher than the corresponding values for Rh and Pt catalysts, 54 % and 45 % respectively (Figures 6g and h). The NMR‐determined solvent retentions coincide well with the quantifications by high‐performance liquid chromatography (HPLC) (Figure S33). Additionally, chemical shift from 3.6 to 5.0 ppm corresponds to the H signal in DOL, and chemical shift from 3.0 to 3.6 ppm are assigned to DME (Figure S2). The integrations of these regions can give quantitative information for respective retention for each DOL and DME solvent. For Co, Rh and Pt catalysts, the DOL retentions are 60 %, 51 %, 41 % and the DME retentions are 69 %, 58 %, 50 %, respectively (Figure S34). The consumption of DOL is more severe than DME due to the ring‐opening reactions of DOL molecules.^[16]^ To quantify the retention of LiTfSI solute, Fourier‐transform infrared spectroscopy with attenuated total reflectance mode (FTIR‐ATR) was used to detect the −CF_3_ group in the cycled electrolyte because the −CF_3_ group comes exclusively from LiTfSI solute. Co‐catalyzed batteries show a retention of 71 % for TfSI^−^ anion, slightly higher than those of 67 % and 65 % for Rh and Pt catalysts (Figure S35). Compared to the severe solvent consumption with lean electrolyte, the consumption of LiTfSI solute is less, coinciding with our previous findings (Figures 1a and b). This comparison confirms superior ability of Co catalyst to restrain solvent loss (Table S2). Therefore, the above results highlight the significance of catalyst‐solvent binding on the battery performance under lean electrolyte conditions.

## Conclusions

The presented comparative study of 3d, 4d, 5d metal catalysts demonstrates that the catalyst‐solvent interaction determines the lean‐electrolyte SRR activity, electrolyte consumption and battery stability. The lean‐electrolyte performance of Li−S batteries can be boosted via strong catalyst‐solvent interactions. This metal‐oxygen interaction between metal catalysts and solvent molecules has been confirmed via a series of synchrotron‐based analysis, in situ spectroscopy and theoretical computations. The strong interaction between Co catalyst and DOL/DME molecules greatly enhance the lean‐electrolyte SRR activity such as higher kinetic current, lower Tafel slope and more Li_2_S deposition. The greater SRR activity of Co catalyst over Rh and Pt is only obvious under lean‐electrolyte conditions, which reveals the significance of strong catalyst‐solvent interaction to boost lean‐electrolyte SRR performance. As a result, the Li−S battery achieves stable cycling, high capacity retention and low electrolyte consumption under lean electrolyte conditions. The role understanding of the catalyst‐solvent interactions should be helpful for the design of electrocatalysts for lean‐electrolyte metal‐sulfur batteries.

## Conflict of interest

The authors declare no conflict of interest.

1

## Supporting information

As a service to our authors and readers, this journal provides supporting information supplied by the authors. Such materials are peer reviewed and may be re‐organized for online delivery, but are not copy‐edited or typeset. Technical support issues arising from supporting information (other than missing files) should be addressed to the authors.

Supporting Information

## Data Availability

The data that support the findings of this study are available from the corresponding author upon reasonable request.
