# Supplementary material for: Unraveling the Catalyst‐Solvent Interactions in Lean‐Electrolyte Sulfur Reduction Electrocatalysis for Li−S Batteries
Source: Angew Chem Int Ed Engl. 2022 Nov 22;61(51):e202213863. doi: 10.1002/anie.202213863 (PMC10099598; doi:10.1002/anie.202213863)
Supplement: Supplementary file 1 — Supporting Information [file ANIE-61-0-s001.pdf]

Angewandte Chemie
Eine Zeitschrift der Gesellschaft Deutscher Chemiker

Supporting Information

Unraveling the Catalyst-Solvent Interactions in Lean-Electrolyte Sulfur Reduction Electrocatalysis for Li–S Batteries

*H. Li, R. Meng, Y. Guo, C. Ye, D. Kong, B. Johannessen, M. Jaroniec, S.-Z. Qiao**

Experimental Procedures

Material Synthesis. Graphite oxide (GO) was synthesized via modified Hummer's method. Typically, 20 mg of GO was ultrasonicated in 20 mL of deionized water for 2 h (1 mg mL^{-1}). 40 mg of L-ascorbic acid sodium-salt was added to the GO suspension under continuous stirring. The suspension was transferred to a glass vial and heated at 70 ^oC for 10 h to obtain a graphene hydrogel. The product was washed with deionized water to remove sodium-salt. 3D graphene hydrogel was soaked in 20 mL of solution containing 238 mg (1 mmol) of cobalt chloride ($CoCl_2 \cdot 6H_2O$) for 24 h. The graphene hydrogel was freeze-dried overnight, and heated at 600 ^oC in 5 vol.% H_2/Ar for 1.5 h. The mixture was repeatedly washed with diluted hydrochloric acid and deionized water to remove larger particles. Nanosized Co metal particles remain on the 3D graphene substrate. Synthesis of other metal nanoparticles follows the same procedure with the same molar concentration (1 mmol) of the chloride-based precursors. Rhodium chloride ($RhCl_3$) and chloroplatinic acid hexahydrate ($H_2PtCl_6 \cdot 6H_2O$) were used to synthesize Rh and Pt nanoparticle catalysts, respectively.

Characterization Techniques. The morphology and structure of samples was characterized by scanning electron microscopy (SEM, Hitachi S4800, Japan). High-resolution STEM images were obtained under a Cs-corrected STEM (FEI Titan Cubed Themis G2 300) operated at 300 kV. Ultraviolet-visible (UV-vis) was carried out using a spectrometer (SHIMADZU UV-2600). X-ray diffraction (XRD) data were collected on a Rigaku Mini Flex 600 X-Ray Diffractometer. X-ray photoelectron spectra (XPS) were measured with the Thermo Fisher Scientific ESCALAB Xi+, Al Kα radiation. Raman spectra were collected with Labram HR Evolution (Horiba Scientific). Fourier-transform infrared spectroscopy with attenuated total reflectance mode (FTIR-ATR) was performed with a Thermo-Fisher Nicolet iS20 equipped with a liquid nitrogen-cooled HgCdTe (MCT) detector using a VeeMax III ATR accessory (Pike Technologies). The electrolyte is extracted and mixed with 500 μL of water for FTIR-ATR tests. High-performance liquid chromatography (HPLC) was performed with Waters LC system while using an Acclaim™ Polar Advantage II column with acetonitrile/water (20%/80%) mobile phase. The electrolyte was mixed with 10 mL of water, and then 100 μL of mixed solution was extracted to mix into 5 mL of water for HPLC tests. The synchrotron-based near-edged X-ray absorption fine structure (NEXAFS) of Co L-edge was performed on the soft X-ray spectroscopy beamline at Australian Synchrotron (Clayton), part of ANSTO. The synchrotron-based extended X-ray absorption fine structure (EXAFS) of Co K-edge was performed on the X-ray absorption beamline at Australian Synchrotron (Clayton), part of ANSTO.

Nuclear Magnetic Resonance (NMR) Analysis. 1,3-dioxolane (DOL) and 1,2-dimethoxyethane (DME) were mixed with a volume ratio of 1:1. 2.87 g of bis(trifluoromethane)sulfonamide lithium salt (LiTfSI) was added into the above 10 mL DOL/DME mixed solvent and stirred overnight into homogeneous solution. Then, 0.276 g of $LiNO_3$ was added into the solution to form the final electrolyte for Li−S batteries. For ^1H and ^{19}F NMR tests, the electrolyte from the cycled cells is extracted with 500 μL of deuterated

dimethyl sulfoxide (d_6-DMSO). Before battery cycling, known amounts of uncycled electrolyte is injected into the coin cells, extracted following the same process and used as a reference (black curves in Figures 1a and 1b). Fluorobenzene is selected as the internal reference for both ^{1}H and ^{19}F tests because its chemical shift is far from those of DOL/DME solvent and TfSI$^-$ anions in both ^{1}H and ^{19}F spectra. 4.7 μL of fluorobenzene is added to the extracted solution for NMR quantifications. The ^{1}H and ^{19}F peaks of fluorobenzene are normalized to 100%. Due to the known content of internal reference, the retention of both DOL/DME solvent or TfSI$^-$ anions in electrolyte can be quantified by comparing the peak areas.

Electrochemical Tests. For Li−S battery testing, the sulfur cathode was prepared by mixing 80 wt.% of S with 20 wt.% of the catalyst. The catalyst/S mixture was ball-milled with LA-133 binder with a mass ratio 90:10. Total sulfur content in the cathode was 72 wt.%. Li−S batteries were assembled with CR2032 coin-cell in an Ar-filled glove box by coupling a piece of Li-metal anode with 1 mol L^{-1} LiTfSI in DOL/DME (1:1 volume ratio) with 0.4 mol L^{-1} $LiNO_3$ as the electrolyte. The cyclic voltammetry (CV) measurements were carried out from 1.7 to 2.8 V with a scan rate of 0.2 mV s^{-1}. For electrochemical test of Li−S batteries including rate and cycling performance, the batteries were galvanostatically charged and discharged at the selected current rates (1C = 1675 mAh g^{-1}) and cycles. The galvanostatic charge-discharge of all the batteries was carried out using Neware battery test system (CT-4008T-5V50mA-164, Shenzhen, China). For the Li_2S nucleation tests, the coin-cell was galvanostatically discharged to 2.06 V at 0.0785 mA, and then potentiostatically discharged at 2.02 V until the current was < 10^{-5} A. For Li_2S nucleation testing, the Li_2S_8 catholyte was prepared by combining sublimed sulfur and Li_2S powder in a molar ratio of 7:1 in LiTfSI/DOL/DME electrolyte under vigorous stirring for 24 h. Carbon-fiber paper (CP) disks with a diameter of 10 mm were used as the substrates to load Co, Rh and Pt catalysts with 1 mg cm^{-2} to assemble the coin-cells. Li-foil was used as the counter electrode. 10 μL of Li_2S_8 (0.25 mol L^{-1}) catholyte was added on the cathode and 5 μL of blank electrolyte on the anode. Therefore, the Li_2S nucleation was tested under lean electrolyte (15 μL) conditions.

Computational details. Computations for this work were carried out using density functional theory (DFT) as implemented in Vienna ab-initio simulation package (VASP) code. Electronic exchange-correlation energy was modeled using the Perdew-Burke-Ernzerhof (PBE) function within a generalized gradient approximation (GGA). The projector-augmented wave (PAW) method was used to describe the ionic cores. For the plane-wave expansion a 450 eV kinetic energy cut-off was used following testing a series of different cut-off energies. A Monkhorst-Pack 2×2×1 k-point grid was used to sample the Brillouin zone. Convergence criterion for the electronic structure iteration was set to 10^{-5} eV, and that for geometry optimizations was 0.02 eV Å^{-1} on force. A Gaussian smearing of 0.1 eV was applied during geometry optimization and for total energy computations.

Binding energy (E_b) was computed by subtracting the energy of substrate and absorbed molecule from the energy of whole system. For example, for DOL on Co metal surface, the binding energy is computed as follows:

$E_b = E\ (\text{Co+DOL}) - E\ (\text{DOL}) - E\ (\text{Co})$

where E (Co+DOL) is the DFT-based total energy of DOL on Co, eV, E (DOL) the energy of individual DOL in the same supercell, eV, and E (Co) the energy of Co, eV. A more negative value means stronger binding interaction.

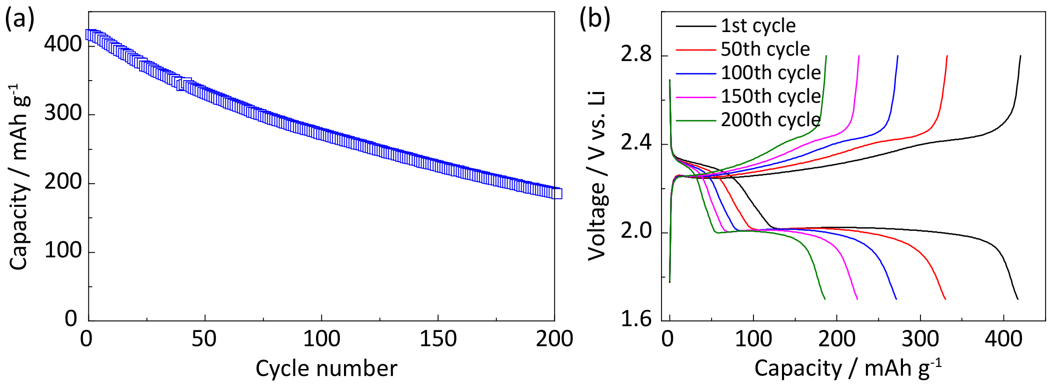

Figure S1. (a) Cycling performance of the Li−S battery, and (b) the corresponding charge-discharge curves at different cycles. The battery was assembled using graphene as sulfur host, and charged-discharged at 0.5 C. The electrolyte/sulfur (E/S) mass ratio is 5.

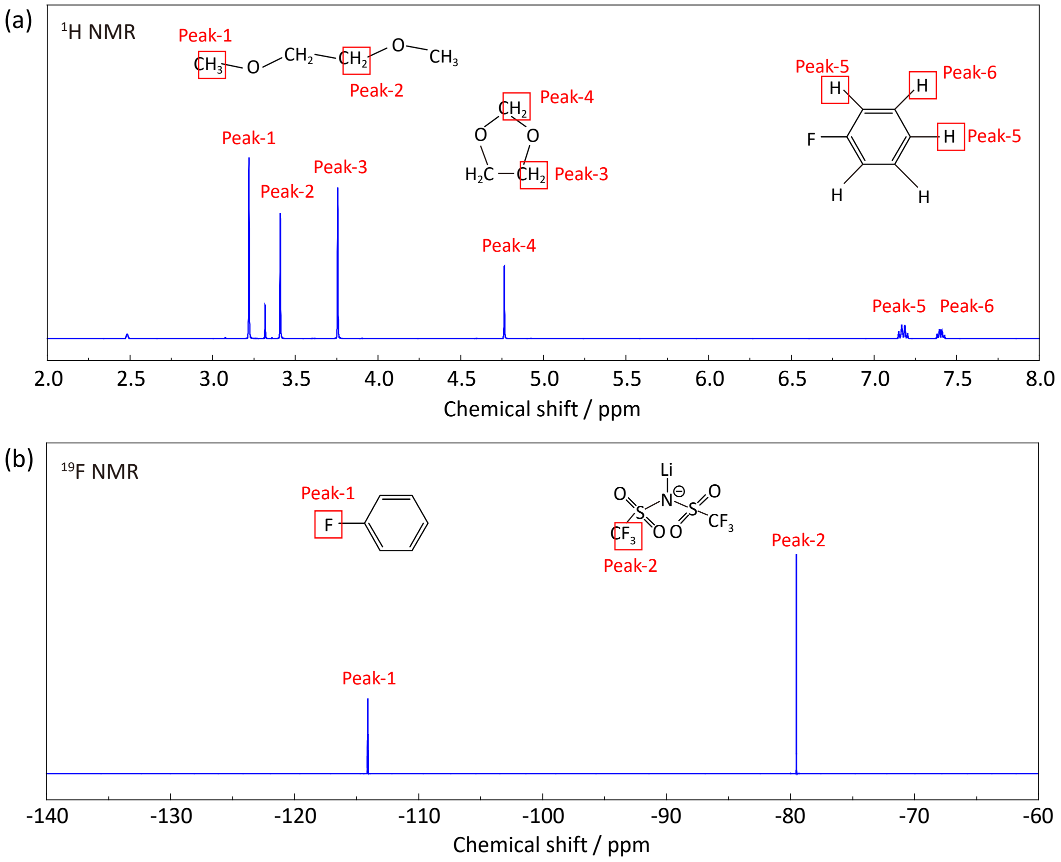

Figure S2. (a) ^{1}H and (b) ^{19}F NMR spectra of DOL, DME and LiTfSI. 0.1 M Fluorobenzene was used as an internal reference to quantify DOL/DME and LiTfSI contents. The insets in these figures present the detailed peak information of H and F with different chemical environments.

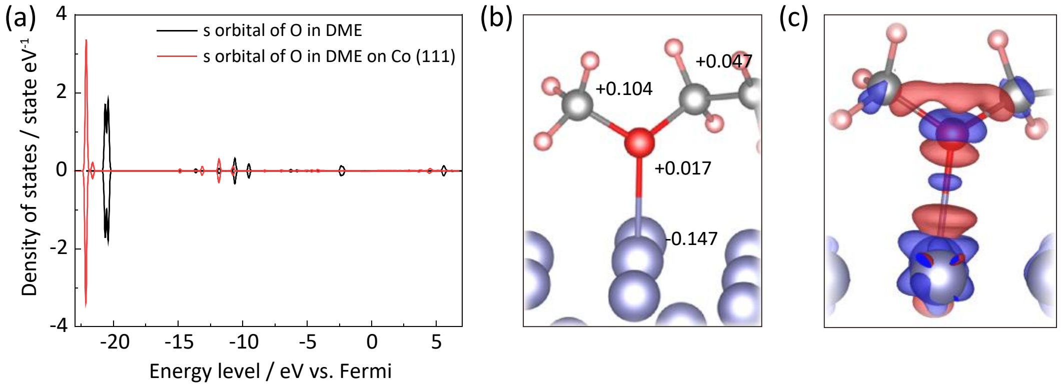

Figure S3. (a) Projected DOS changes of O s-orbital for pure DME and DME adsorbed on Co (111); (b) Bader charge analysis of DME on Co (111); (c) Charge-difference figure of DME on Co (111) wherein red indicates electron accumulation and blue denotes electron depletion.

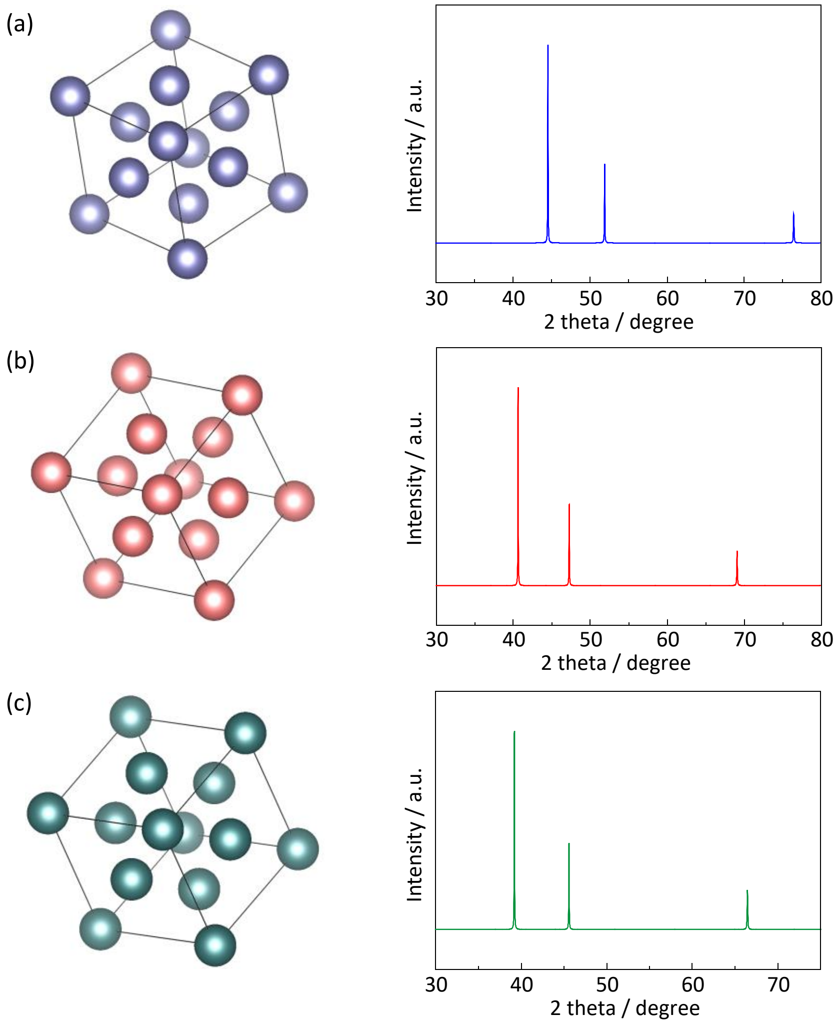

Figure S4. Crystal structures of (a) Co, (b) Rh, (c) Pt metals and the corresponding simulated XRD patterns. The results agree with the XRD results in Figure 1f.

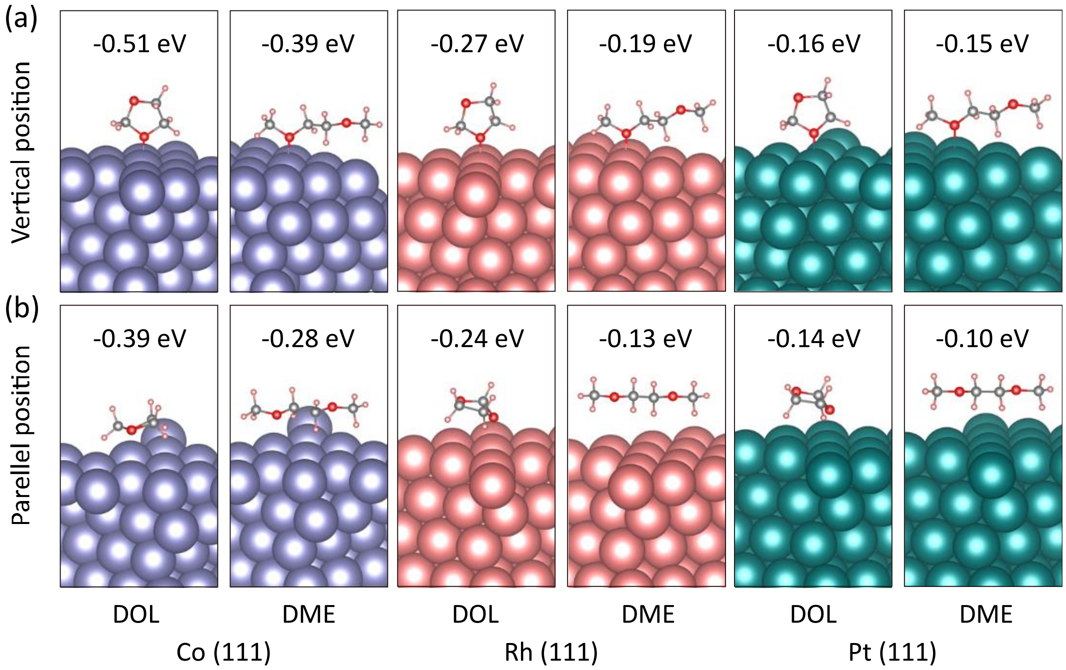

Figure S5. The optimized structures of DOL and DME molecules with (a) vertical position and (b) parallel position on Co (111), Rh (111) and Pt (111). The binding energies with vertical positions are higher than those with parallel positions. This suggests that the solvent molecules prefer to adsorb on the catalyst surface by vertical alignment. In the parallel case, the repulsion between surface metal atoms and edged hydrogen atoms leads to a weak binding.

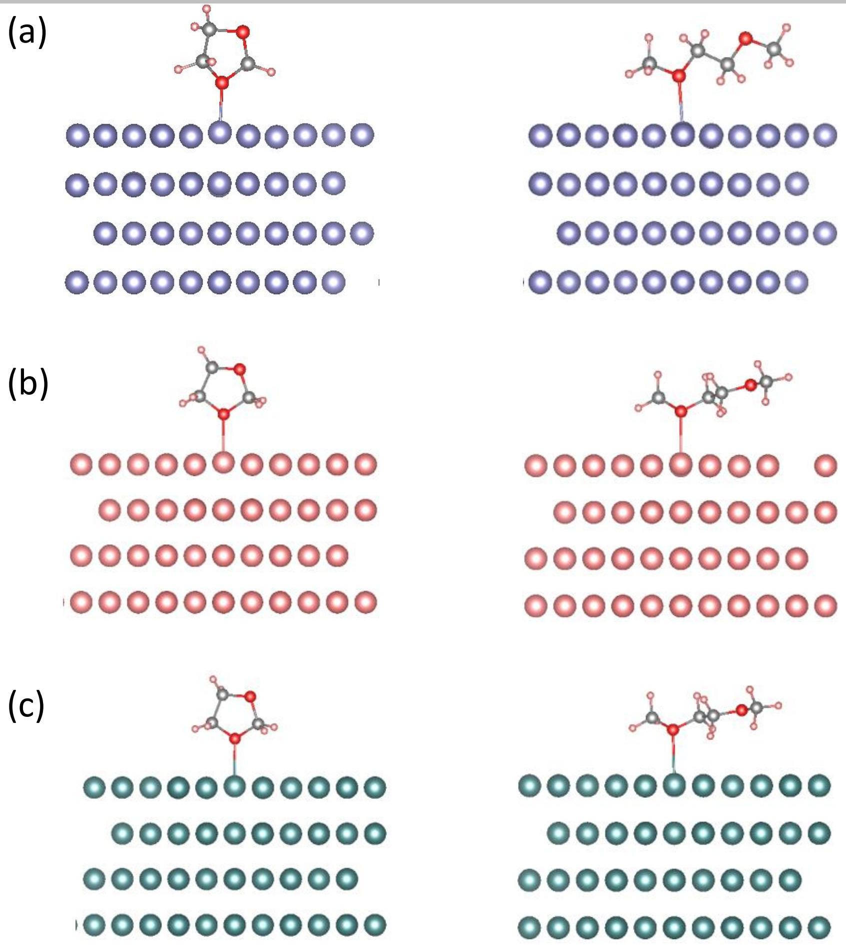

Figure S6. Optimized structures of DOL and DME on (a) Co (111), (b) Rh (111) and (c) Pt (111). Purple, light red, green atoms present Co, Rh, Pt, respectively. Grey, red, and pink atoms denote carbon, oxygen and hydrogen, respectively. Metal−oxygen bonds are identified based on these optimized models.

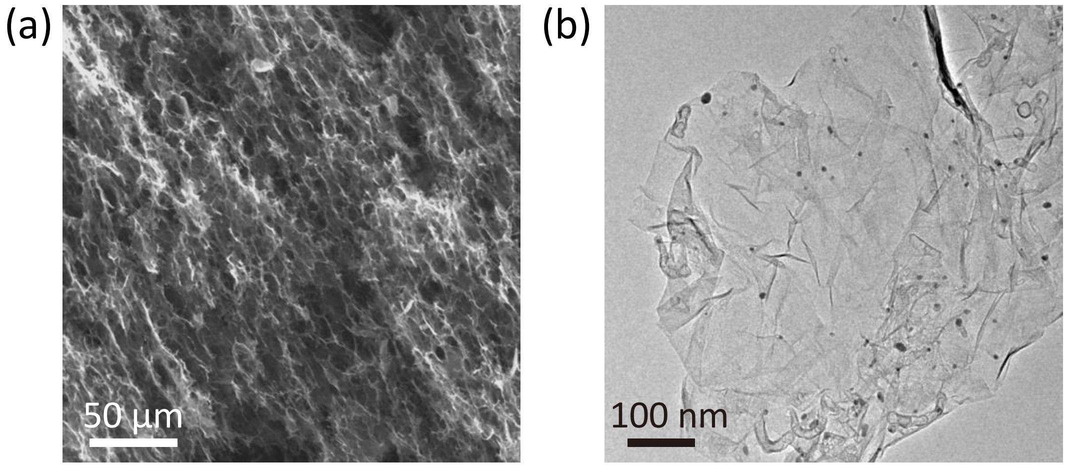

Figure S7. Microscopic images of graphene substrate and Co nanoparticles. (a) SEM image of graphene substrate. 3D interconnected graphene network is observed in this image; (b) TEM image of Co nanoparticles on graphene substrate.

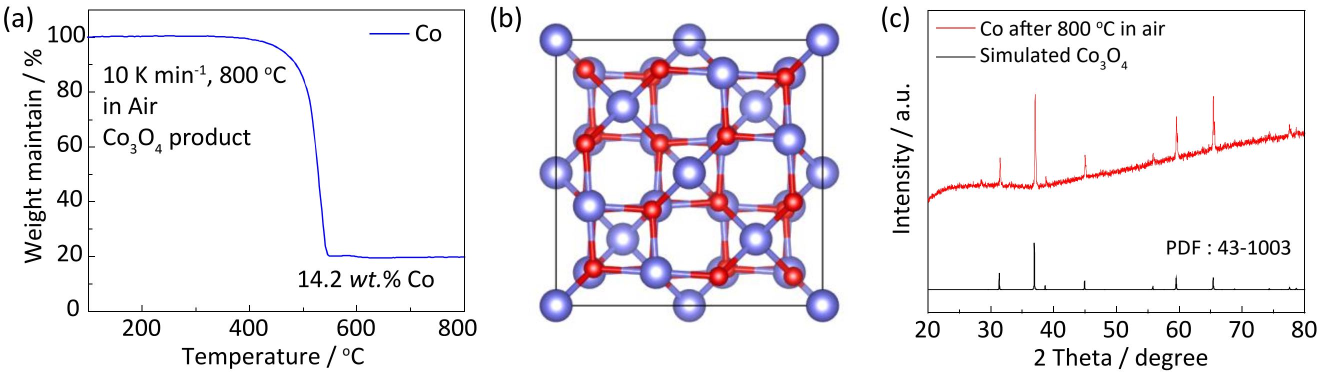

Figure S8. (a) Thermogravimetric analysis (TGA) of synthesized Co catalyst on graphene substrate in air; (b) The crystal structure of Co_3O_4 and (c) the experimental and simulated XRD patterns of Co_3O_4. This confirms the Co_3O_4 product after annealing Co nanoparticle at 800 °C in air. The Co content is 14.2 *wt.*%.

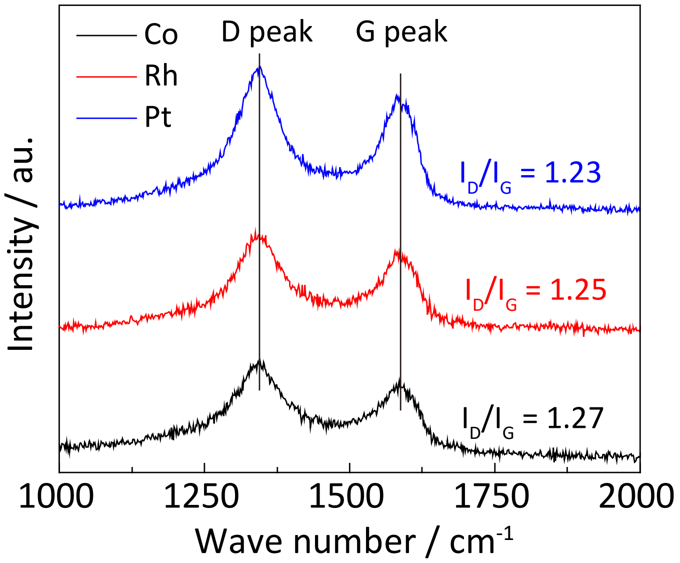

Figure S9. Raman spectra of graphene substrates with different metal loading. The I_D/I_G value does not change significantly. This suggests that the loading of Co, Rh and Pt does not alter the properties of graphene substrate.

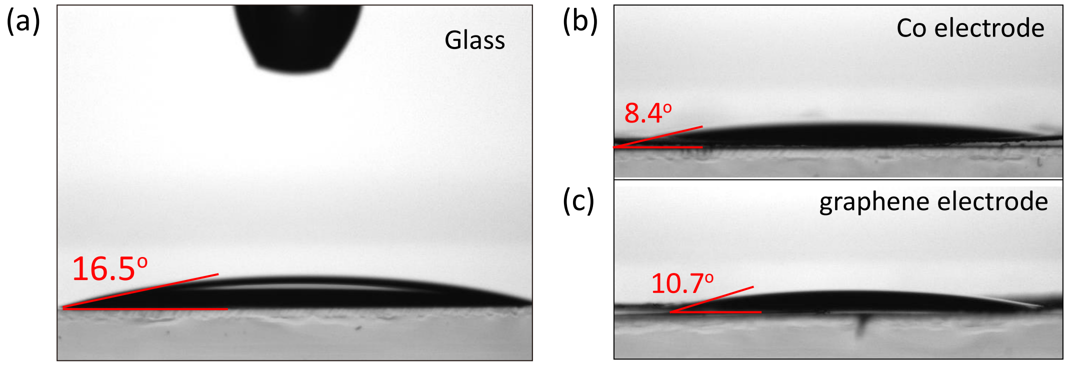

Figure S10. The contact angle of DOL/DME droplet on (a) glass, (b) Co-catalyst electrode, and (c) graphene electrode. The lower contact angle on the Co-catalyst electrode confirms higher affinity of electrolyte solvent toward Co surface.

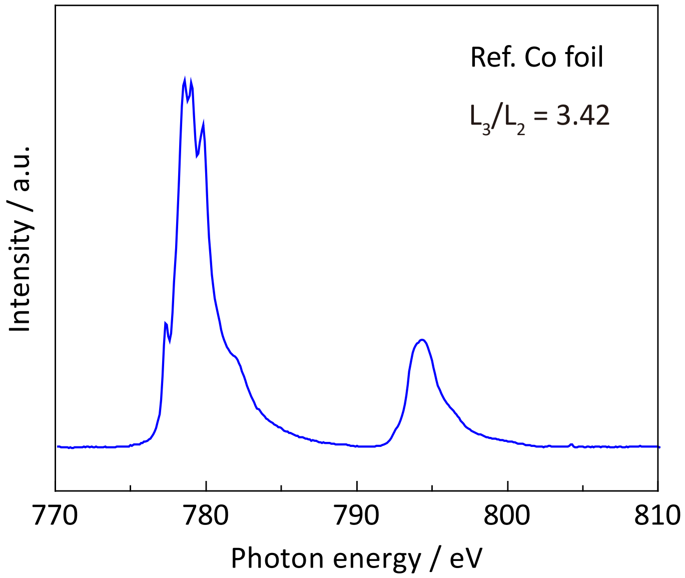

Figure S11. The Co L-edge near-edge X-ray adsorption fine structure (NEXAFS) of reference Co foil. The peak ratio of L_3/L_2 is 3.42.

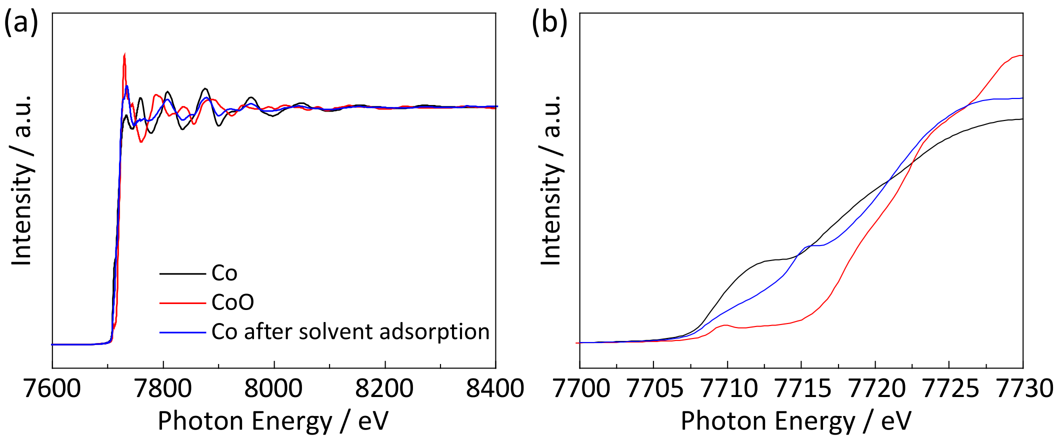

Figure S12. (a) The Co K-edge extended X-ray adsorption fine structures (EXAFS) of synthesized Co catalyst, CoO and Co catalyst after solvent adsorption; (b) The enlarged figure shows the white-line ranges of these samples.

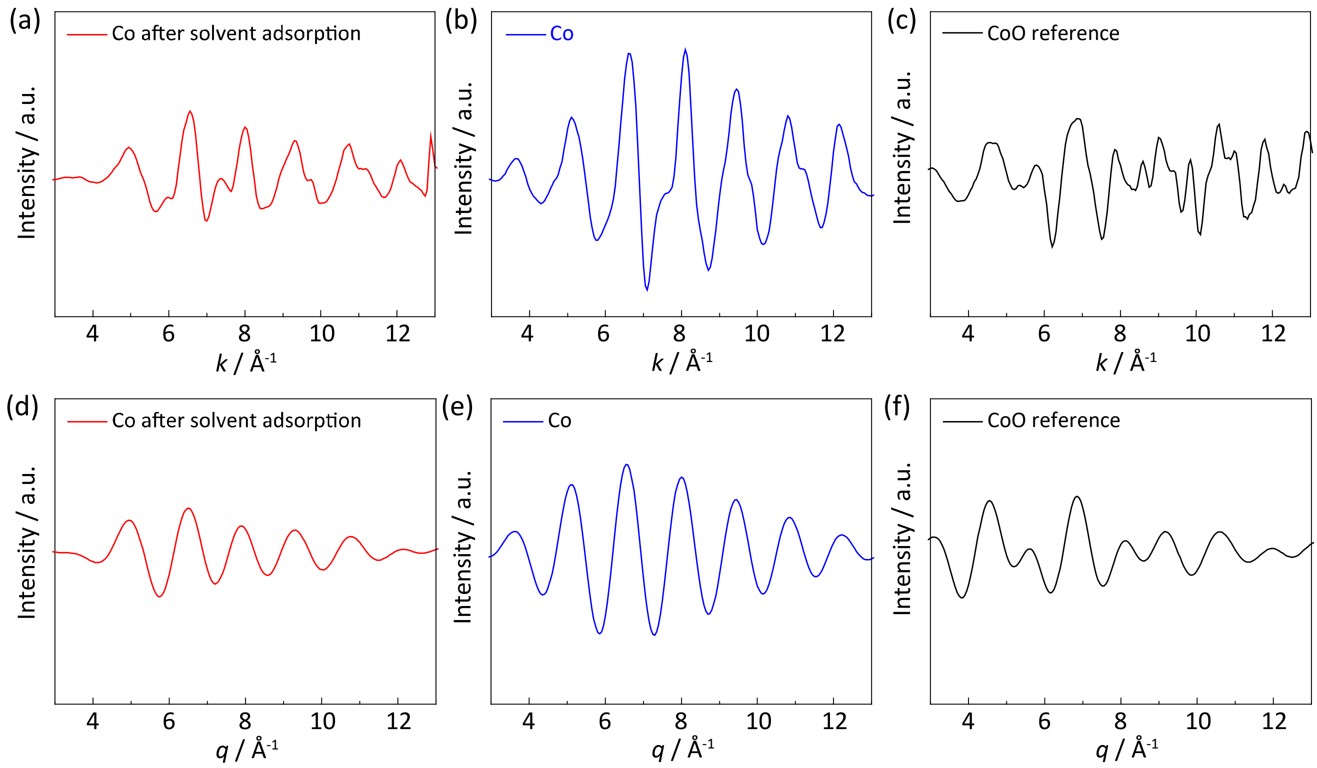

Figure S13. The k-space curves of (a) Co after solvent adsorption, (b) Co and (c) CoO reference; and the transformed q-space data of (d) Co after solvent adsorption, (e) Co and (f) CoO reference.

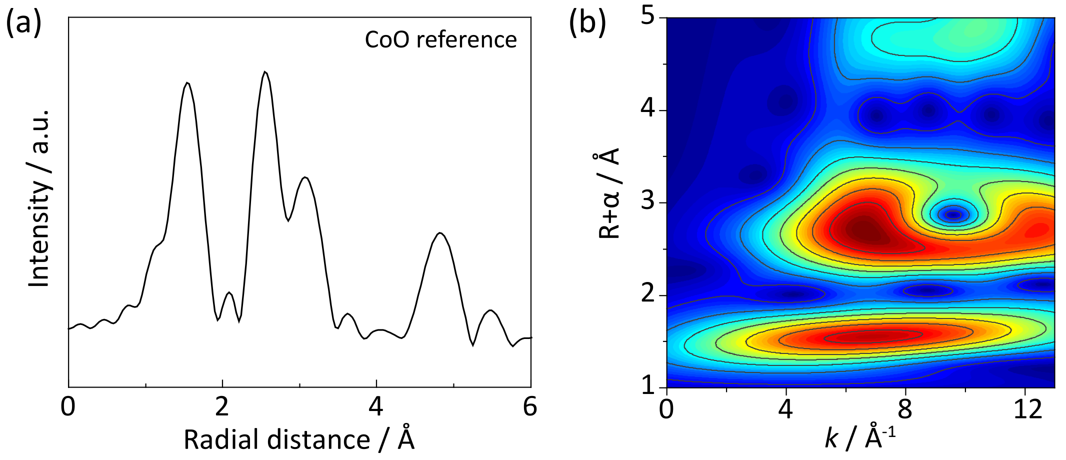

Figure S14. (a) The radial distance in R-space of CoO reference and (b) the corresponding wavelet-transformed contour pattern.

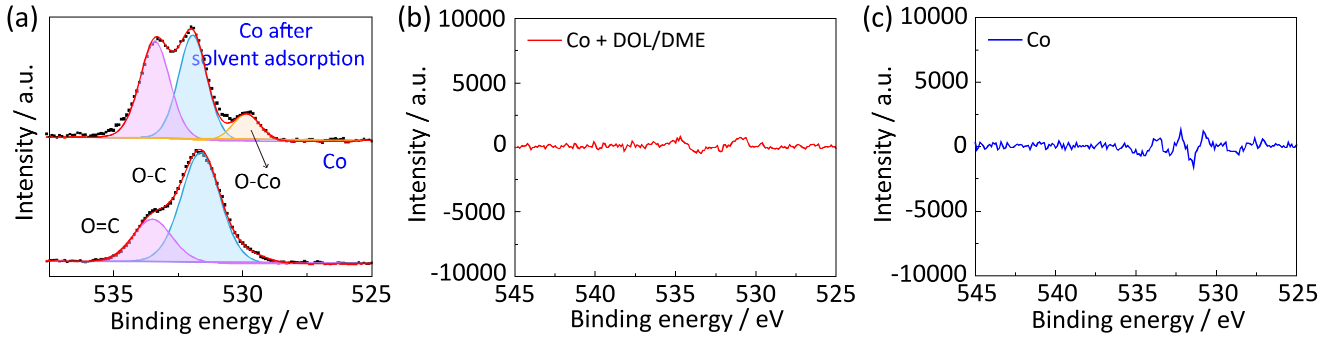

Figure S15. (a) XPS spectra of Co catalyst and Co after DOL/DME adsorption; and (b, c) the corresponding subtracted curves between the raw and fitting data for the spectra.

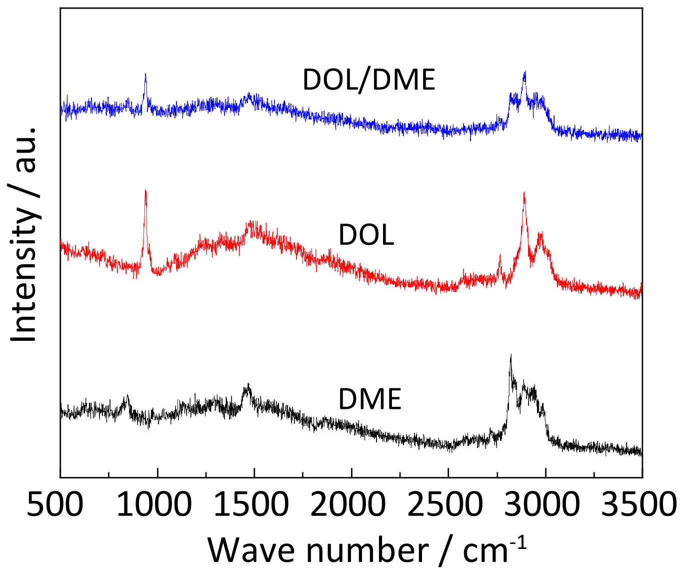

Figure S16. Raman spectra of pristine DOL, DME and DOL/DME mixed solvent.

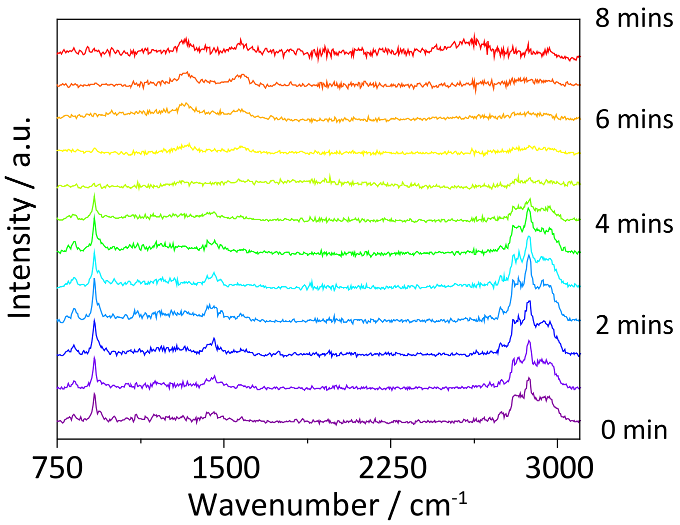

Figure S17. The time-dependent Raman spectra of DOL/DME solvent on pristine graphene substrate.

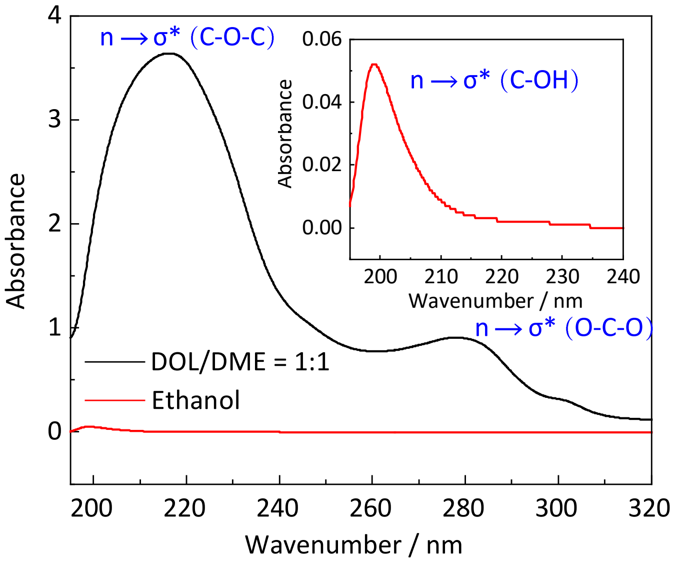

Figure S18. UV-vis absorption curves of DOL/DME and ethanol. The absorption peaks for C-OH, C-O-C and O-C-O functional groups are ~200 nm, ~210 nm and ~280 nm respectively. Besides, the peak for ethanol is very weak (inset Figure), and therefore ethanol is selected to dissolve different amounts of DOL/DME.

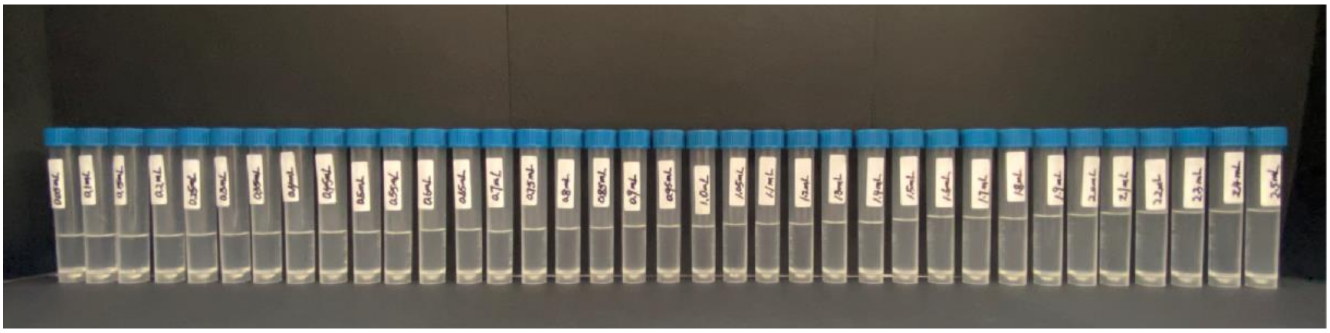

Figure S19. Digital photograph of DOL/DME in ethanol with different volume ratios. These standard solutions were used to obtain the relation between the UV-vis peak intensity and the volume concentration of DOL/DME mixed solvent.

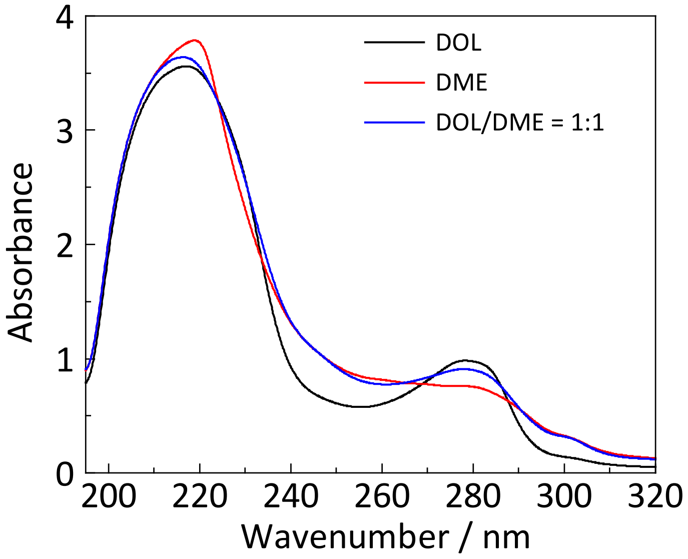

Figure S20. The UV-vis spectra of pure DOL, pure DME and DOL/DME mixed solvent.

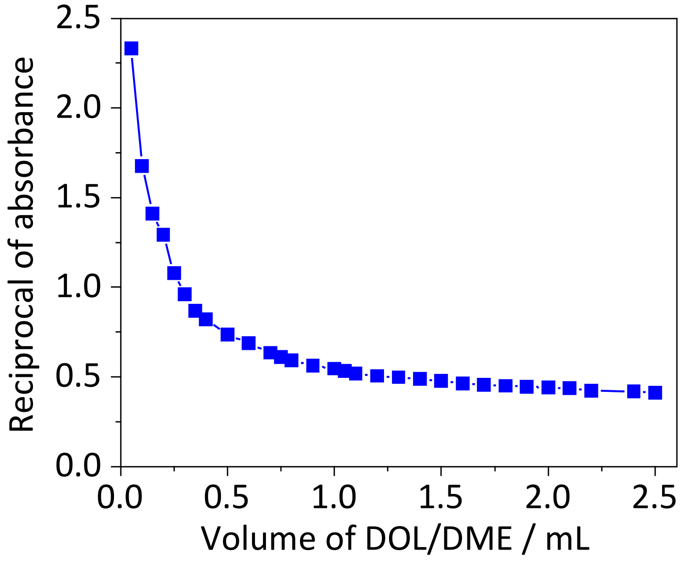

Figure S21. The relationship between the reciprocal of UV-vis absorbance and the volume of DOL/DME solvent.

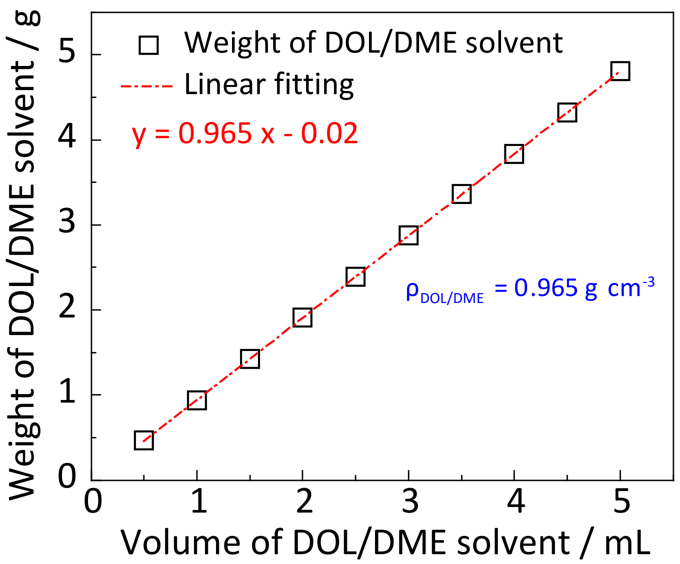

Figure S22. The relation between the weight and volume of DOL/DME mixed solvent. The density is calculated as 0.965 g cm^{-3} with the linear fitting.

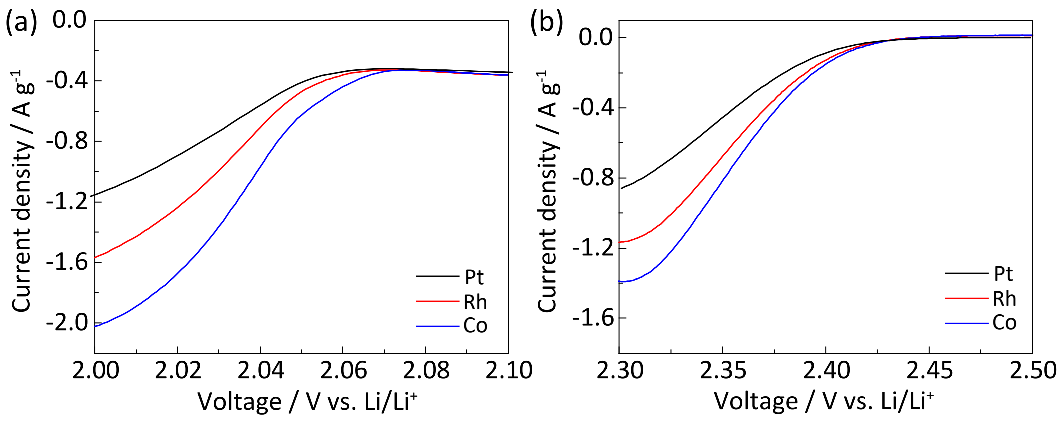

Figure S23. Enlarged CV curve of Figure 4a at regions of (a) high-voltage catalytic reduction from sulfur to polysulfides (2.50 to 2.30 V) and (b) low-voltage reduction from polysulfides to Li_2S (2.10 to 2.00 V).

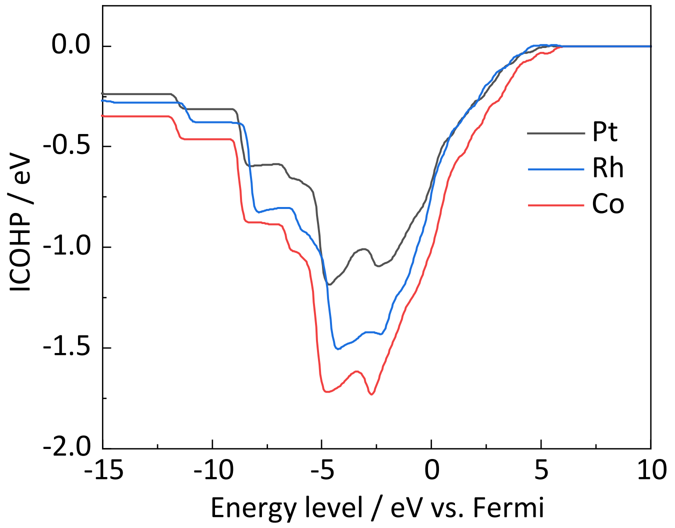

Figure S24. Integrated crystal orbital Hamilton population (ICOHP) between oxygen atom among DOL and the surface metal atoms. A more negative value at Fermi level suggests a stronger bonding between metal catalyst and solvent molecules.

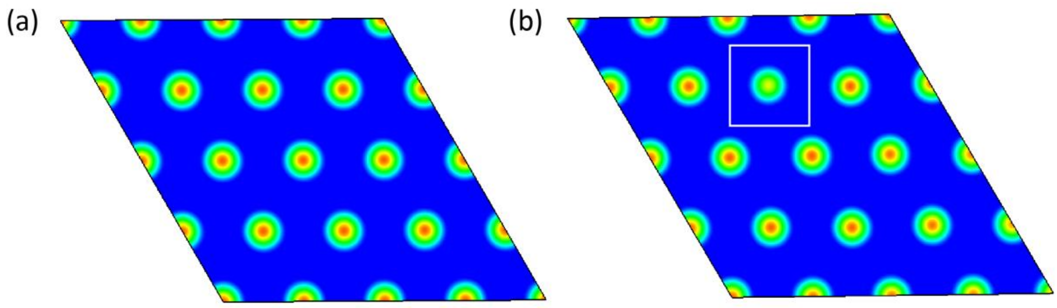

Figure S25. The charge density of (a) Co (111) surface and (b) Co (111) surface after DOL adsorption. The decreased density indicates the electron transfer from Co to DOL molecule (as marked by the while square in b).

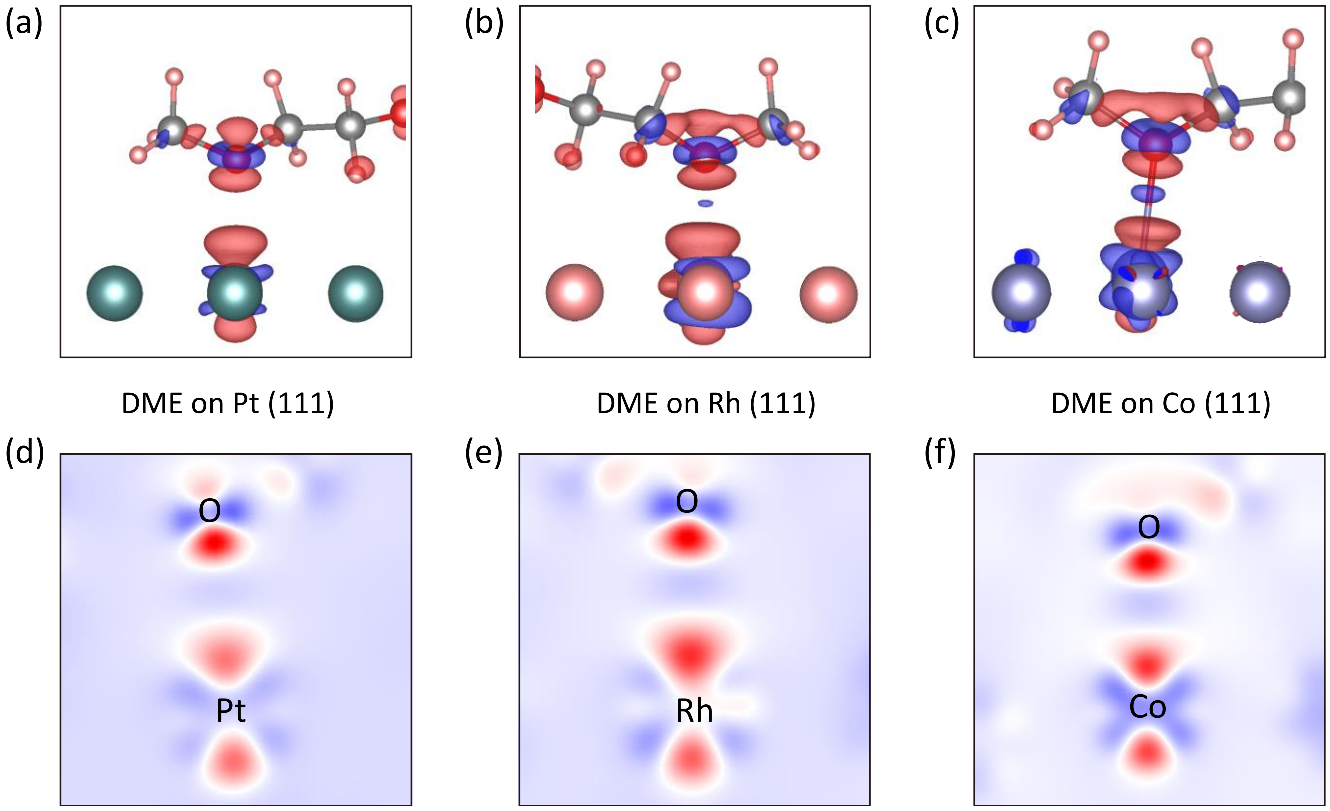

Figure S26. Charge differences of DME molecule on metal surfaces. 3D charge-difference figures of DME on (a) Pt (111), (b) Rh (111) and (c) Co (111); 2D sliced charge-difference figures of DME on (d) Pt (111), (e) Rh (111) and (f) Co (111). Red indicates electron accumulation and blue denotes electron depletion.

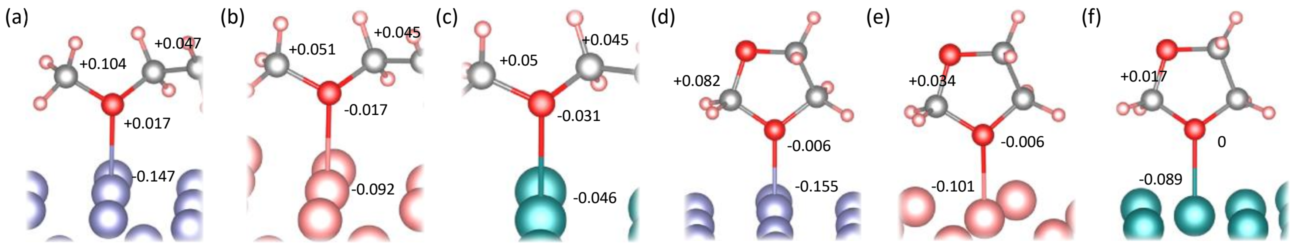

Figure S27. Bader charge analysis of adsorbed DOL/DME molecules on different metal surfaces. DME molecule on (a) Co (111), (b) Rh (111) and (c) Pt (111); DOL molecule on (d) Co (111), (e) Rh (111) and (f) Pt (111). The selected Co atom shows a higher value of electron depletion for both DOL and DME molecules. This confirms its strong binding with the solvent molecules.

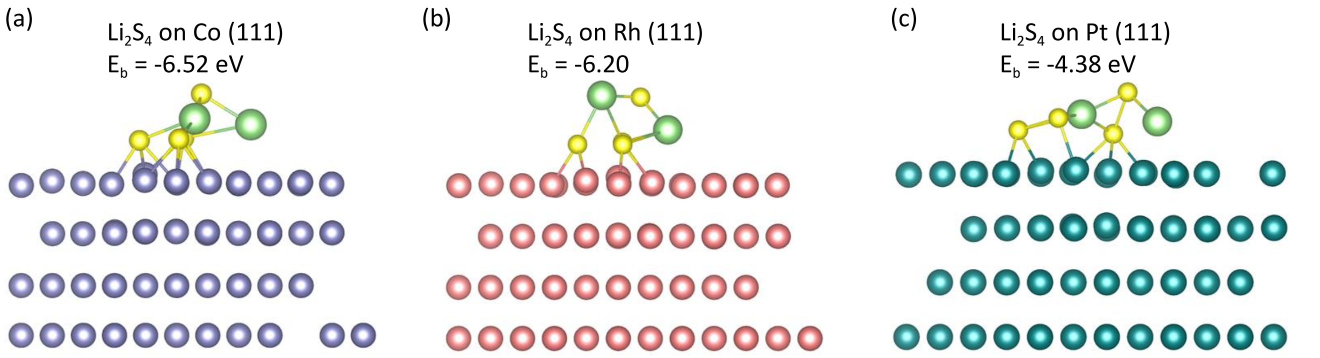

Figure S28. The optimized structures of Li_2S_4 on (a) Co (111), (b) Rh (111) and (c) Pt (111). The binding energies of $Li_2S_4^*$ on Co (111), Rh (111) and Pt (111) surface are -6.52 eV, -6.20 eV and -4.38 eV respectively. Too strong binding energy of Co would lead to a lower catalytic activity (**Figure 6a**).

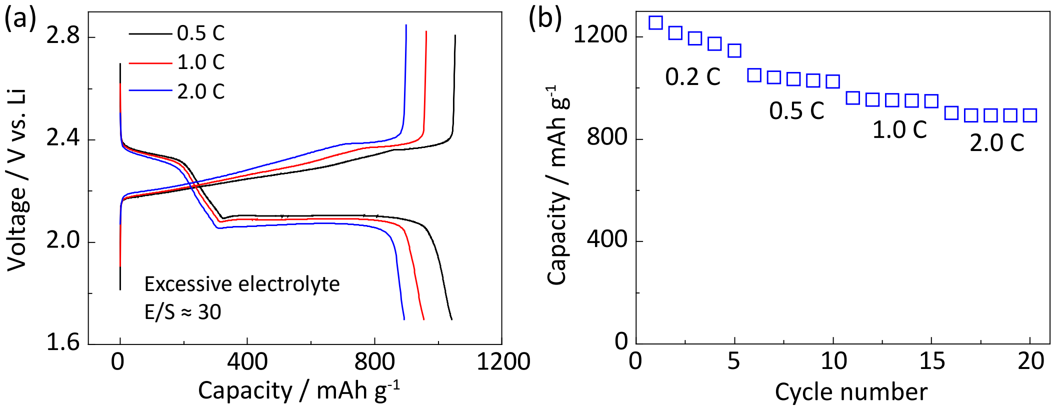

Figure S29. (a) Charge-discharge curves of Li−S batteries with Pt catalyst, and (b) capacity retention at different rates ranging from 0.2 to 2.0 C. The areal mass loading of sulfur is ≈ 0.5 mg cm^{-2} with excessive electrolyte. Pt demonstrates excellent battery performance with flooded electrolyte; however, its performance dramatically degrades under lean-electrolyte conditions.

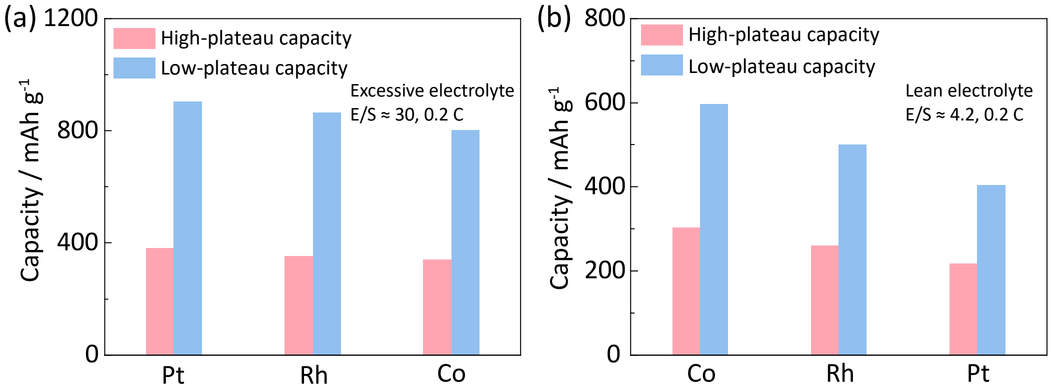

Figure S30. High-plateau and low-plateau capacity for sulfur cathodes using Co, Rh and Pt catalysts with (a) excessive electrolyte and (b) lean electrolyte. The capacity dramatically changes under lean electrolyte conditions.

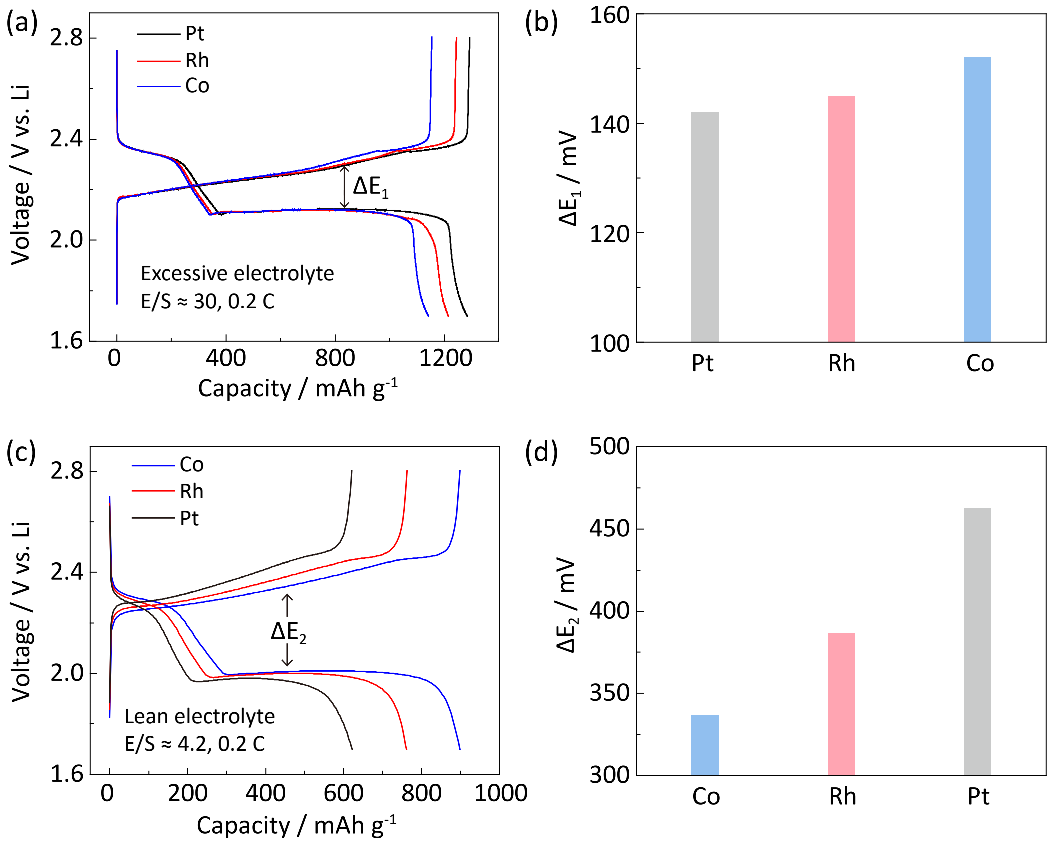

Figure S31. Charge-discharge curves of Li−S batteries using Co, Rh, Pt catalysts (a) with excessive electrolyte and (c) lean electrolyte; The corresponding charge-discharge overpotentials (b) with excessive electrolyte and (d) lean electrolyte. The charge-discharge overpotentials (ΔE) present a significant change under lean electrolyte conditions.

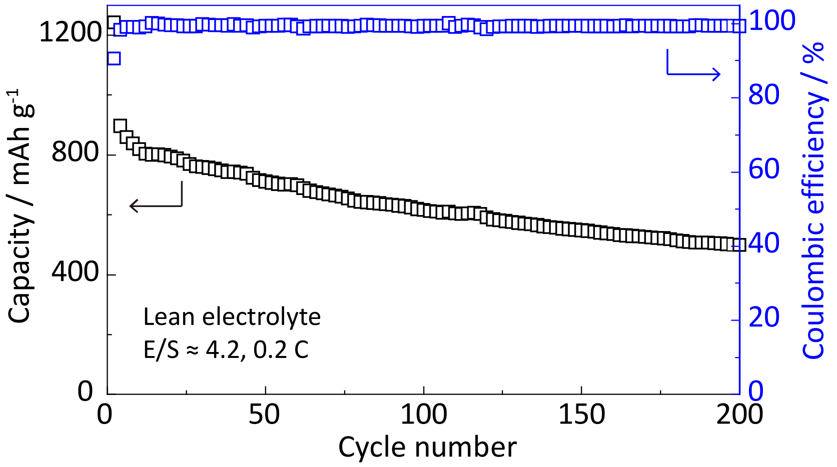

Figure S32. Cycling performance of Li−S battery with Co catalyst under lean electrolyte conditions shows a stable cycling with the Coulombic efficiency nearly 100%.

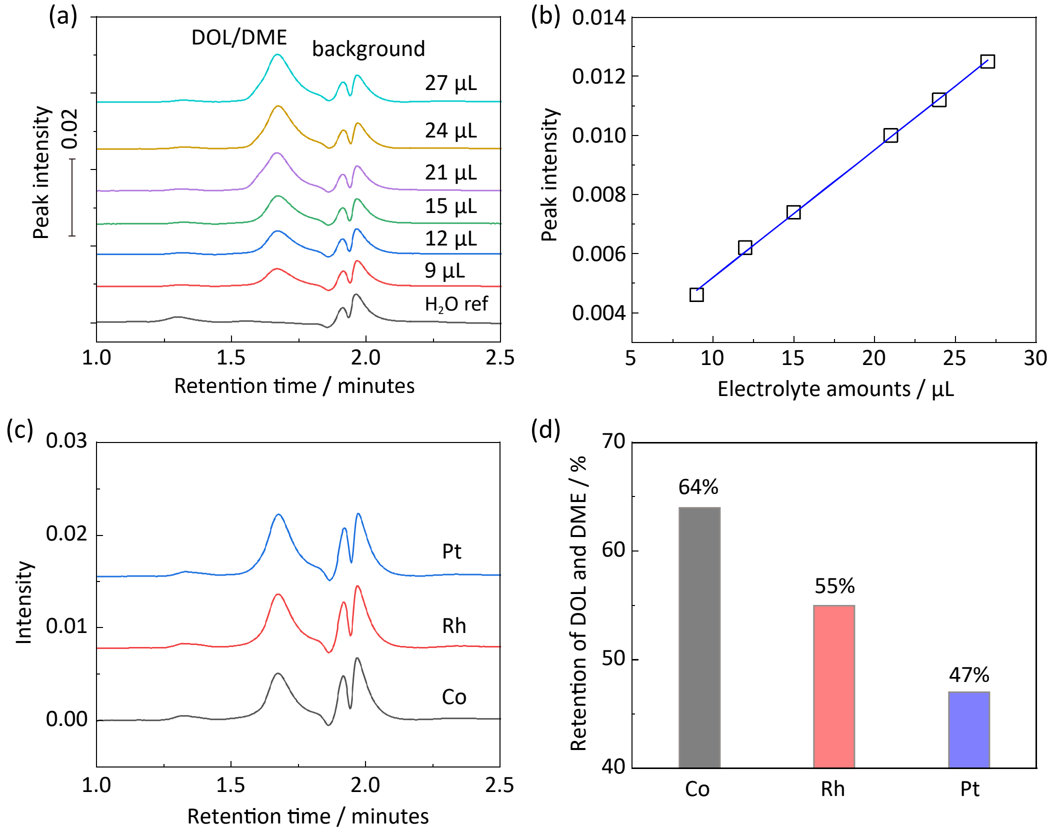

Figure S33. HPLC results to determine the retention of DOL and DME mixed solvent for the cycled electrolytes. (a) HPLC curves for the mixed solution with different electrolyte injection; (b) Standard relation between known amounts of electrolyte volume and HPLC peak intensity; (c) HPLC spectra for the cycled electrolyte with Co, Rh and Pt catalysts; (d) The retention of DOL and DME with Co, Rh and Pt catalysts.

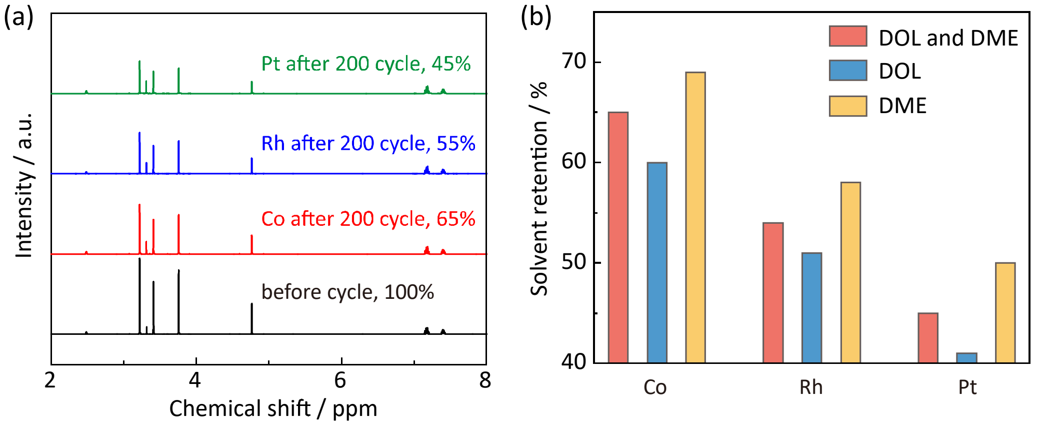

Figure S34. (a) ^{1}H NMR spectra of electrolytes extracted from un-cycled battery, and Co, Rh, Pt catalyzed Li−S batteries after 200 cycles. (b) the overall DOL and DME retentions are 65%, 54% and 45% for Co, Rh and Pt catalysts respectively. Among these catalysts, the DOL retentions are 60%, 51%, 41% and the DME retentions are 69%, 58%, 50%, respectively.

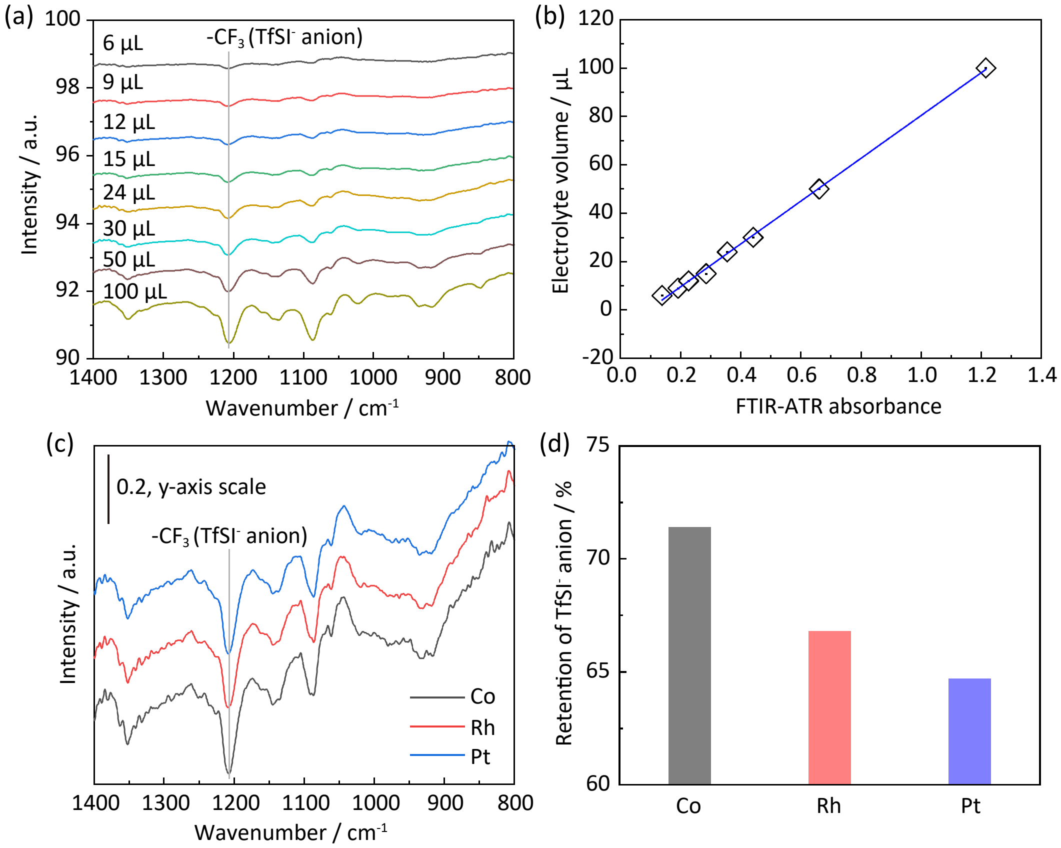

Figure S35. FTIR-ATR results to determine the retention of TfSI$^-$ anion for the cycled electrolytes. (a) FRIR-ATR spectra for the mixed solution with different electrolyte injection in 500 μL of water; (b) Standard relation between known amounts of TfSI$^-$ anion (electrolyte volume) and FRIR-ATR peak intensity; (c) FTIR-ATR spectra for the cycled electrolyte with Co, Rh and Pt catalysts; (d) The retention of TfSI$^-$ anion with Co, Rh and Pt catalysts.

Table S1 Performance comparison of lean-electrolyte Li-S batteries reported in this work and elsewhere

Reported Catalysts	Electrolyte dosage / μL mg^{-1}	Sulfur loading / mg cm^{-2}	Current density	Capacity (flooded electrolyte) / mAh g^{-1}	Capacity with (lean electrolyte) / mAh g^{-1}	Capacity retention	References
MoS_2 with $EV(ClO_4)_2$ mediator	5.0	5.6	0.5 mA cm^{-2}	~1320	~1022	77%	1
MoS_2	5.0	4.0	0.2 C	~700	~500	71%	2
Co-NC	8.3	3.8	0.2 C	~920	~450	49%	3
LDH	6.0	5.5	0.2 C	~1000	~727	73%	4
HPP	7.0	8.1	0.01 C	~1378	~981	72%	5
In_2O_3	4.6	5.9	0.1	~1320	~950	72%	6
Fe-Ni alloy	4.5	4.1	0.05 C	~1000 (0.2 C)	~600	60%	7
$NiCl_2$	5.0	N/A	0.5 C	~820	~630	77%	8
Ni_3FeN	4.7	4.8	0.2 C	~1150	~500	43.4%	9
$NiSe_2$	6.0	8.8	0.2 C	~1110	~852	77%	10
Fe-C_3N_4	3.8	N/A	0.2 C	~1270	~800	63%	11
MoP	4.0	6.0	0.8 mA cm^{-2}	N/A	~880	N/A	12
MgB_2	6.5	8.3	0.2 C	1250 (0.05 C)	~850	68%	13
MoB	7.0	6.1	0.2 C	~1200	~800	67%	14
Black P	6.5	4.0	0.1 C	~1280	~800	63%	15
Co	4.2	5.0	0.2 C	1142	900	79%	This work

Table S2 Retention of different electrolyte components after battery cycling

	LiTfSI retention (FTIR-ATR)	DOL and DME retention (HPLC)	DOL and DME retention (NMR)	DOL retention (NMR)	DME retention (NMR)
Co catalyst	71%	64%	65%	60%	69%
Rh catalyst	67%	55%	54%	51%	58%
Pt catalyst	65%	47%	45%	41%	50%

Supplementary References

[1] H. Ye, J. Sun, X. F. Lim, Y. Zhao, J. Y. Lee, *Energy Storage Mater.* **2021**, *38*, 338-343.

[2] Q. Wu, Z. Yao, X. Zhou, J. Xu, F. Cao, C. Li, *ACS Nano* **2020**, *14*, 3365-3377.

[3] H. Gao, S. Ning, Y. Zhou, S. Men, X. Kang, *Chem. Eng. J.* **2021**, *408*, 127323.

[4] C. Li, Y. Zhao, Y. Zhang, D. Luo, J. Liu, T. Wang, W. Gao, H. Li, X. Wang, *Chem. Eng. J.* **2021**, *417*, 129248.

[5] Z. Ye, Y. Jiang, L. Li, F. Wu, R. Chen, *Adv. Mater.* **2020**, *32*, 2002168.

[6] W. Hua, H. Li, C. Pei, J. Xia, Y. Sun, C. Zhang, W. Lv, Y. Tao, Y. Jiao, B. Zhang, S. Z. Qiao, Y. Wan, Q. H. Yang, *Adv. Mater.* **2021**, *33*, 2101006.

[7] J. He, A. Bhargav, A. Manthiram, *ACS Nano* **2021**, *15*, 8583-8591.

[8] C. Luo, X. Liang, Y. Sun, W. Lv, Y. Sun, Z. Lu, W. Hua, H. Yang, R. Wang, C. Yan, J. Li, Y. Wan, Q.-H. Yang, *Energy Storage Mater.* **2020**, *33*, 290-297.

[9] M. Zhao, H.-J. Peng, Z.-W. Zhang, B.-Q. Li, X. Chen, J. Xie, X. Chen, J.-Y. Wei, Q. Zhang, J.-Q. Huang, *Angew. Chem. Int. Ed.* **2019**, *58*, 3779-3783.

[10] A. H. Shao, X.-X. Zhang, Q.-S. Zhang, X. Li, Y. Wu, Z. Zhang, J. Yu, Z.-Y. Yang, *ACS Applied Energy Mater.* **2021**, *4*, 3431-3438.

[11] C. Lu, Y. Chen, Y. Yang, X. Chen, *Nano letters* **2020**, *20*, 5522-5530.

[12] Y. Yang, Y. Zhong, Q. Shi, Z. Wang, K. Sun, H. Wang, *Angew. Chem. Int. Ed.* **2018**, *57*, 15549-15552.

[13] Q. Pang, C. Y. Kwok, D. Kundu, X. Liang, L. F. Nazar, *Joule* **2019**, *3*, 136-148.

[14] J. He, A. Bhargav, A. Manthiram, *Adv. Mater.* **2020**, *32*, 2004741.

[15] Z.-L. Xu, S. Lin, N. Onofrio, L. Zhou, F. Shi, W. Lu, K. Kang, Q. Zhang, S. P. Lau, *Nat. Commun.* **2018**, *9*, 4164.

Author Contributions

S.-Z. Q. conceived and supervised this research; H. L. designed and carried out experiments and DFT computations; R. M. carried out the synthesis of metal catalysts and electrochemical tests; Y. G. carried out XPS tests; C. Y. assisted with NEXAFS and EXAFS tests; D. K. captured the TEM images; B. J. carried out the synchrotron EXAFS test and related data analyses; S.-Z. Q. and M. J. corrected and edited the manuscript. All authors discussed results and commented on the manuscript.
